# Guided search for hybrid systems based on coarse-grained space abstractions

**DOI:** 10.1007/s10009-015-0393-y

**Published:** 2015-08-07

**Authors:** Sergiy Bogomolov, Alexandre Donzé, Goran Frehse, Radu Grosu, Taylor T. Johnson, Hamed Ladan, Andreas Podelski, Martin Wehrle

**Affiliations:** IST Austria, Klosterneuburg, Austria; University of Freiburg, Freiburg, Germany; University of California, Berkeley, USA; Université Joseph Fourier Grenoble 1 – Verimag, Grenoble, France; Vienna University of Technology, Vienna, Austria; University of Texas at Arlington, Arlington, USA; University of Basel, Basel, Switzerland

**Keywords:** Hybrid system, Reachability analysis, Guided search, Heuristics, Pattern databases

## Abstract

Hybrid systems represent an important and powerful formalism for modeling real-world applications such as embedded systems. A verification tool like SpaceEx is based on the exploration of a symbolic search space (the *region space*). As a verification tool, it is typically optimized towards proving the absence of errors. In some settings, e.g., when the verification tool is employed in a feedback-directed design cycle, one would like to have the option to call a version that is optimized towards finding an error trajectory in the region space. A recent approach in this direction is based on *guided search*. Guided search relies on a cost function that indicates which states are promising to be explored, and preferably explores more promising states first. In this paper, we propose an abstraction-based cost function based on *coarse-grained space abstractions* for guiding the reachability analysis. For this purpose, a suitable abstraction technique that exploits the flexible granularity of modern reachability analysis algorithms is introduced. The new cost function is an effective extension of pattern database approaches that have been successfully applied in other areas. The approach has been implemented in the SpaceEx model checker. The evaluation shows its practical potential.

## Introduction

Hybrid systems are extended finite automata whose discrete states correspond to the various modes of continuous dynamics a system may exhibit, and whose transitions express the switching logic between these modes [1]. Hybrid systems have been used to model and to analyze various types of embedded systems [5, 9, 19, 20, 34, 35, 41]. A hybrid system is considered safe if a given set of bad states cannot be reached from the initial states. Hence, reachability analysis is a main concern for hybrid systems. Since the reachability analysis of hybrid systems is in general undecidable [1], modern reachability analysis tools such as SpaceEx [23] resort to semi-decision procedures based on over-approximation techniques [16, 23]. In this paper, we explore the utility of guided search in order to improve the efficiency of such techniques.

**Fig. 1:**
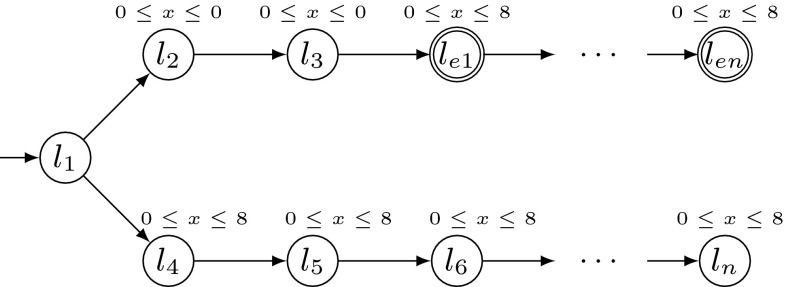
A motivating example

Guided search is an approach that has recently found much attention for finding errors in large systems [14, 30]. As suggested by the name, guided search performs a search in the state space of a given system. In contrast to standard search methods like breadth-first or depth-first search (DFS), the search is guided by a cost function that estimates the search effort to reach an error state from the current state. This information is exploited by preferably exploring states with lower estimated costs. If accurate cost functions are applied, the search effort can significantly be reduced compared to uninformed search. Obviously, the cost function therefore plays a key role within the setting of guided search, as it should be as accurate as possible on the one hand, and as cheap to compute as possible on the other. Cost functions that have been proposed in the literature are mostly based on *abstractions* of the original system. An important class of abstraction-based cost functions is based on *pattern databases (PDBs)*. PDBs have originally been proposed in the area of Artificial Intelligence [17] and also have successfully been applied to model checking discrete and timed systems [29, 30, 37, 42]. Roughly speaking, a PDB is a data structure that contains abstract states together with abstract cost values based on an abstraction of the original system. During the concrete search, concrete states *s* are mapped to corresponding abstract states in the PDB, and the corresponding abstract cost values are used to estimate the costs of *s*. Overall, PDBs have demonstrated to be powerful for finding errors in different formalisms. The open question is if guided search can be applied equally successfully to finding errors in hybrid systems.

A first approach in this direction [14] is to estimate the cost of a symbolic state based on the Euclidean distance from its continuous part to a given set of error states. This approach appears to be best suited for systems whose behavior is strongly influenced by the (continuous) differential equations. However, it suffers from the fact that discrete information like mode switches is completely ignored, which can lead to arbitrary degeneration of the search. To see this, consider the example presented in Fig. 1. It shows a simple hybrid system with one continuous variable which obeys the differential equation $\dot{x}=1$ in every location (differential equations are omitted in the figure). The error states are given by the locations $l_{e1},\dots ,l_{en}$ and invariants $0\le x\le 8$. In this example, the box-based distance heuristic wrongly explores the whole lower branch first (where no error state is reachable) because it only relies on the continuous information given by the invariants. More precisely, for the box-based distance heuristic, the invariants suggest that the costs of the “lower” states are equal to 0, whereas the costs of the “upper” states are estimated to be equal to 4 (i. e., equal to the distance of the centers of the bounding boxes of the invariants).

To overcome these limitations, we introduce an abstraction-based cost function for hybrid systems which is motivated by PDBs. In contrast to the box-based approach based on Euclidean distances, this cost function is able to properly reflect the discrete part of the system. Compared to the “classical” discrete setting, the investigation of PDBs for hybrid systems becomes more difficult for several reasons. First, hybrid systems typically feature both discrete and continuous variables with complex dependencies and interactions. Therefore, the question arises how to compute a suitable (accurate) abstraction of the original system. Second, computations for symbolic successors and inclusion checks become more expensive than for discrete or timed systems—can these computations be performed or approximated efficiently to get an overall efficient PDB approach as well? In this paper, we provide answers to these questions, leading to an efficient guided search approach for hybrid systems. In particular, we introduce an abstraction technique leveraging properties of the set representations used in modern reachability algorithms. By simply using coarser parameters for the explicit representation, we obtain suitable and cheap *coarse-grained space abstractions* for the behaviors of a given hybrid system. Furthermore, we adapt the idea of *partial* PDBs, which has been originally proposed for solving discrete search problems [7], to the setting of hybrid systems in order to reduce the size and computation time of “classical” PDBs. Our implementation in the SpaceEx tool [23] shows the practical potential.

The remainder of the paper is organized as follows. After introducing the necessary background for this work in Sect. 2, we present our PDB approach for hybrid systems in Sect. 3. This is followed by a discussion about related work in Sect. 4. Afterwards, we present our experimental evaluation in Sect. 5. Finally, we conclude the paper in Sect. 6.

## Preliminaries

In this section, we introduce the preliminaries that are needed for this work.

### Notations

We consider models that can be represented by hybrid systems. A hybrid system is formally defined as follows.

#### **Definition 1**

(*Hybrid System*) A *hybrid system* is a tuple $\mathcal {H}=( Loc , Var , Init , Flow , Trans , Inv )$ defining

- The finite set of locations $Loc$,
- The set of continuous variables $Var =\{x_1, \ldots , x_n\}$ from $\mathbb {R}^n$,
- The initial condition, given by the constraint $Init (\ell )\subset \mathbb {R}^n$ for each location $\ell$,
- For each location $\ell$, a relation called $Flow(\ell )$ over the variables and their derivatives. We assume $Flow (\ell )$ to be of the form
  $$\begin{aligned} \dot{x}(t) = Ax(t)+u(t),\quad u(t) \in \mathcal {U}, \end{aligned}$$
  where $x(t)\in \mathbb {R}^n$, *A* is a real-valued $n \times n$ matrix and $\mathcal {U}\subseteq \mathbb {R}^n$ is a closed and bounded convex set,
- The discrete transition relation, given by a set $Trans$ of discrete transitions; a discrete transition is formally defined as a tuple $(\ell ,g,\xi ,\ell ')$ defining
  - The source location $\ell$ and the target location $\ell '$,
  - The guard, given by a linear constraint $g$,
  - The update, given by an affine mapping $\xi$, and
- The invariant $Inv (\ell ) \subset \mathbb {R}^n$ for each location $\ell$.

The semantics of a hybrid system $\mathcal {H}$ is defined as follows. A *state* of $\mathcal {H}$ is a tuple $(\ell ,\mathbf {x})$, which consists of a location $\ell \in Loc$ and a point $\mathbf {x}\in \mathbb {R}^n$. More formally, $\mathbf {x}$ is a valuation of the continuous variables in $Var$. For the following definitions, let ${\mathcal {T}}=[0,\varDelta ]$ be an interval for some $\varDelta \ge 0$. A *trajectory* of $\mathcal {H}$ from state $s=(\ell ,\mathbf {x})$ to state $s'=(\ell ',\mathbf {x}')$ is defined by a tuple $\rho =(L, \mathbf {X})$, where $L:{\mathcal {T}}\rightarrow Loc$ and $\mathbf {X}:{\mathcal {T}}\rightarrow \mathbb {R}^n$ are functions that define for each time point in ${\mathcal {T}}$ the location and values of the continuous variables, respectively. Furthermore, we will use the following terminology for a given trajectory $\rho$. A sequence of time points where location switches happen in $\rho$ is denoted by $(\tau _i)_{i=0\ldots k}\in {\mathcal {T}}^{k+1}$. In this case, we define the *length* of $\rho$ as $|\tau |=k$. Trajectories $\rho =(L, \mathbf {X})$ (and the corresponding sequence $(\tau _i)_{i=0\ldots k}$) have to satisfy the following conditions:

- $\tau _0=0$, $\tau _i<\tau _{i+1}$, and $\tau _k=\varDelta$ – the sequence of switching points increases, starts with 0 and ends with $\varDelta$
- $L(0)=\ell$, $\mathbf {X}(0)=\mathbf {x}$, $L(\varDelta )=\ell '$, $\mathbf {X}(\varDelta )=\mathbf {x}'$ – the trajectory starts in $s=(\ell ,\mathbf {x})$ and ends in $s'=(\ell ',\mathbf {x}')$
- $\forall i\;\forall t\in [\tau _i,\tau _{i+1}): L(t)=L(\tau _i)$ – the location is not changed during the continuous evolution
- $\forall i\; \forall t \in [\tau _i,\tau _{i+1}): \;\; (\mathbf {X}(t),\dot{\mathbf {X}}(t))\in Flow (L(\tau _i))$, i.e., $\dot{\mathbf {X}}(t) = A\mathbf {X}(t)+u(t)$ holds and thus the continuous evolution is consistent with the differential equations of the corresponding location
- $\forall i\; \forall t\in [\tau _i,\tau _{i+1}):\; \mathbf {X}(t) \in Inv (L(\tau _i))$ – the continuous evolution is consistent with the corresponding invariants
- $\forall i\; \exists (L(\tau _i),g,\xi ,L(\tau _{i+1}))\in Trans : \mathbf {X}_{end}(i) = \lim _{\tau \rightarrow \tau _{i+1}^-}\mathbf {X}(\tau )\wedge \mathbf {X}_{end}(i)\in ~g \wedge \mathbf {X}(\tau _{i+1})=\xi (\mathbf {X}_{end}(i))$ – every continuous transition is followed by a discrete one, $\mathbf {X}_{end}(i)$ defines the values of continuous variables right before the discrete transition at the time moment $\tau _{i+1}$, whereas $\mathbf {X}_{start}(i)=\mathbf {X}(\tau _i)$ denotes the values of continuous variables right after the switch at the time moment $\tau _i$.

A state $s'$ is *reachable* from state *s* if there exists a trajectory from *s* to $s'$.

In the following, we mostly refer to *symbolic states*. A symbolic state $s=(\ell ,\mathcal {R})$ is defined as a tuple, where $\ell \in Loc$, and $\mathcal {R}$ is a convex and bounded set consisting of points $\mathbf {x}\in \mathbb {R}^n$. The continuous part $\mathcal {R}$ of a symbolic state is also called *region*. The symbolic state space of $\mathcal {H}$ is called the *region space*. The initial set of states $\mathcal {S}_{ init }$ of $\mathcal {H}$ is defined as $\bigcup _{\ell }(\ell , Init (\ell ))$. The reachable state space $\mathrm {Reach}(\mathcal {H})$ of $\mathcal {H}$ is defined as the set of symbolic states that are reachable from an initial state in $\mathcal {S}_{ init }$, where the definition of reachability is extended accordingly for symbolic states.

In this paper, we assume there is a given set of symbolic bad states $\mathcal {S}_{ bad }$ that violate a given property. Our goal is to find a sequence of symbolic states which contains a trajectory from $\mathcal {S}_{ init }$ to a symbolic *error state*, where a symbolic error state $s_e$ has the property that there is a symbolic bad state in $\mathcal {S}_{ bad }$ that agrees with $s_e$ on the discrete part, and that has a non-empty intersection with $s_e$ on the continuous part. A trajectory that starts in a symbolic state *s* and leads to a symbolic error state is called an *error trajectory* $\rho _e(s)$.

### Symbolic states representation

The representation of symbolic states plays a crucial role for the reachability analysis of hybrid systems. As outlined in the previous section, a symbolic state consists of a discrete location and a continuous region. The handling of continuous regions within the reachability analysis poses a special challenge as a number of operations on polyhedra (such as linear maps, Minkowski sum, and convex hull computation) need to be performed efficiently in practice. The LGG scenario [33] which is implemented in SpaceEx [23] relies on two main ingredients for this purpose: support functions [10] and template polyhedra. In the following, we will describe them in more detail.

The support function $\rho _\mathcal {R}(\ell )$ of a region $\mathcal {R}$ with respect to the direction $\ell \in \mathbb {R}^n$ is defined as follows:

$$\begin{aligned} \rho _\mathcal {R}(\ell ) = \max \limits _{x\in \mathcal {R}} \ell \cdot x. \end{aligned}$$

We can represent an arbitrary convex closed set $\mathcal {R}$ by using support functions in the following way:

$$\begin{aligned} \mathcal {R}= \mathop {\bigcap }_{\ell \in \mathbb {R}^n} \{ x \> | \> \ell \cdot x \le \rho _{\mathcal {R}}(\ell ) \}. \end{aligned}$$

The representation based on support functions allows for efficiently computing all the above-mentioned polyhedra operations, hence reachability algorithms in turn benefit from this representation.

**Fig. 2:**
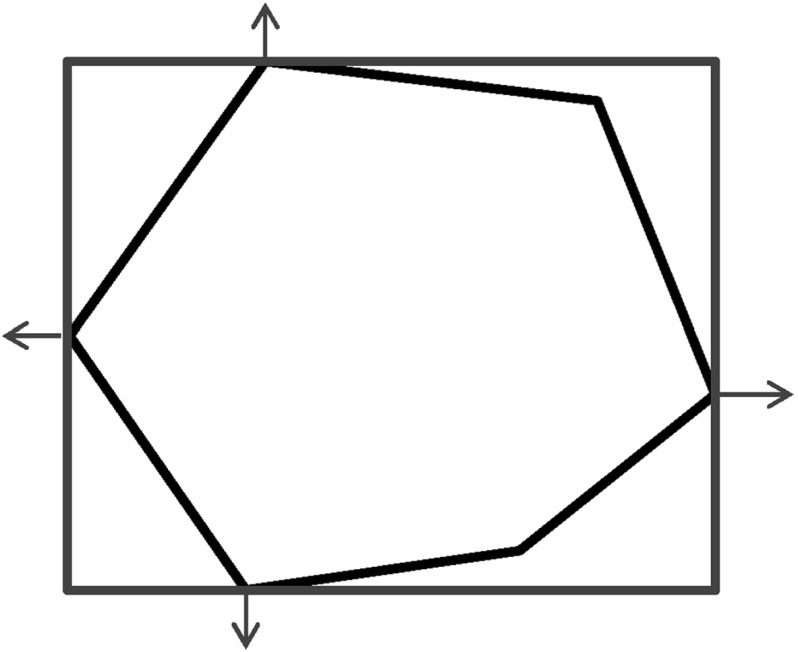
Region representation using box directions

**Fig. 3:**
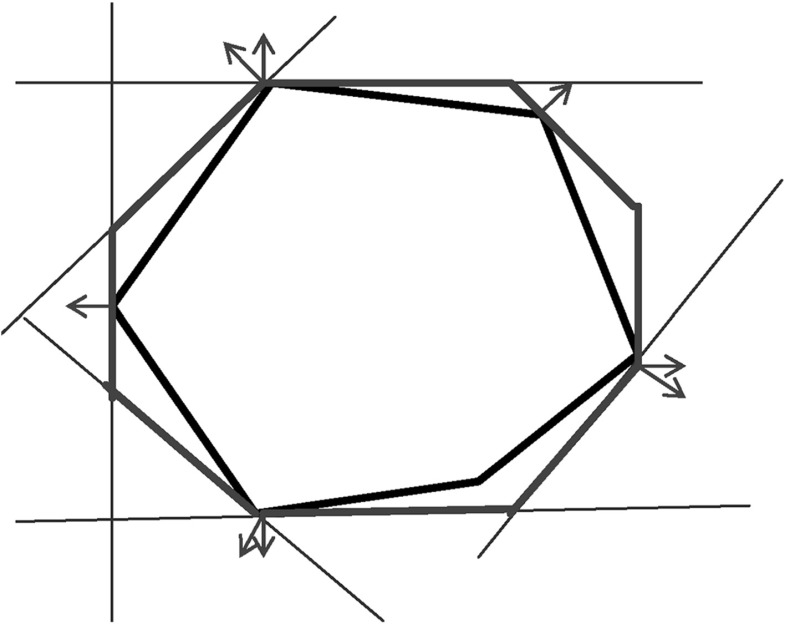
Region representation using octagonal directions

As the consideration of an infinite number of directions is clearly infeasible from the computational point of view, SpaceEx also makes use of a continuous set representation derived from the support functions: *template* polyhedra. In this setting, we *predefine* from the very beginning a set of directions taken into account in course of the reachability analysis. In other words, a user provides a set of directions $D=\{\ell _1,\ldots ,\ell _m\}$ used for the reachability analysis. Based on *D*, the region $\mathcal {R}$ can be over-approximated by the following polyhedron:

$$\begin{aligned} \mathcal {R}_D = \left\{ x \in \mathbb {R}^n\> | \> \mathop {\bigwedge }_{\ell _i \in D} \ell _i \cdot x \le \rho _\mathcal {R}(\ell _i) \right\} . \end{aligned}$$

SpaceEx supports a number of predefined direction sets such as, box directions (directions parallel to axes; see Fig. 2) and octagonal directions (the union of directions parallel to axes and diagonal ones; see Fig. 3). Obviously, by increasing the number of considered directions, we can improve the approximation precision.

In the rest of this section, we briefly recapitulate the computation of continuous successors for a given symbolic state, i.e., the states which are reachable according to the continuous dynamics. As the continuous post operator does not change the discrete part of a symbolic state, we consider only the continuous region of a symbolic state.

The LGG scenario computes the continuous successors only for a finite time horizon. Therefore, we use a *time-bounded version* of the reachable region $\mathrm {Reach}_{t_1,t_2}(\mathcal {R})$ for a given starting region $\mathcal {R}\subseteq \mathbb {R}^n$, dynamics $\dot{x}(t)= Ax(t)+u(t), u(t)\in \mathcal {U}$ (*) and a time interval $[t_1,t_2] \subseteq \mathbb {R}^{\ge 0}$:

$$\begin{aligned} \mathrm {Reach}_{t_1,t_2}(\mathcal {R}) = \{ x(\tau ) \> | \>&t_1 \le \tau \le t_2, x(0) \in \mathcal {R}, \\&x(\tau ) \text{ is } \text{ the } \text{ solution } \text{ of } \text{(*) }\}. \end{aligned}$$

**Fig. 4:**
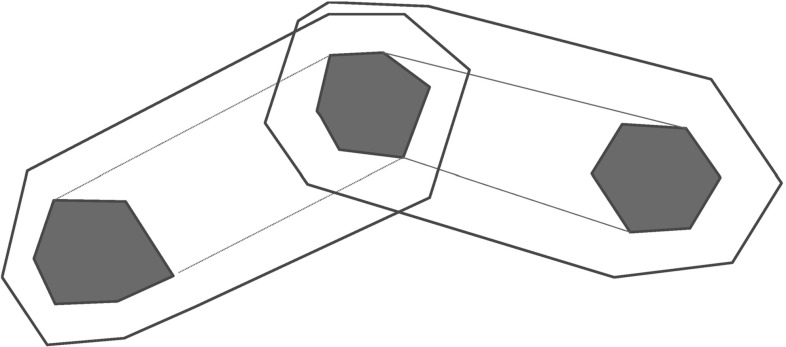
Region representation using a large sampling time

SpaceEx performs an over-approximating time-bounded reachability analysis of $\mathrm {Reach}_{0,T}(\mathcal {R})$, where $T \in \mathbb {R}^{\ge 0}$ is a user-provided *time horizon*. In more detail, as the reachability analysis of hybrid systems is generally undecidable, SpaceEx over-approximates the successor regions by iteratively computing over-approximations based on *discretizing* the time up to the time horizon: First, the time interval [0, *T*] is partitioned in a number of small time intervals $[\delta _i,\delta _{i+1}]$, where $\delta _i = i \cdot T_\delta$ ($i=0,\ldots ,N-1$) and $T_\delta = T / N$ ($N \in \mathbb {N}$) is a user-provided *sampling time*. Second, given this partitioning, SpaceEx covers the exact reachability set with the sequence $\varOmega _i~\subseteq ~\mathbb {R}^n, i=0,\ldots ,N-1$, where $\varOmega _i$ defines the over-approximation of the states reachable within the time interval $[\delta _i,\delta _{i+1}]$. In other words, the following inclusion holds:

$$\begin{aligned} \mathrm {Reach}_{0,T} (Init) \subseteq \mathop {\bigcup }_{i=0}^{N-1} \varOmega _i. \end{aligned}$$

**Fig. 5:**
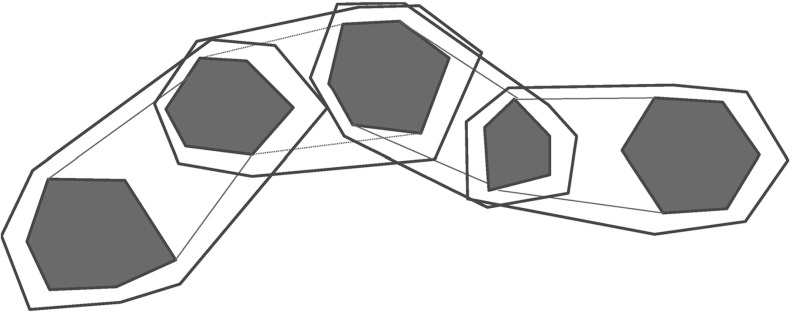
Region representation using a small sampling time

The set $\varOmega _{i+1}$ can be expressed in terms of the “predecessor set” $\varOmega _i$ by using a linear map and Minkowski sum. Therefore, we only need to provide a routine to compute $\varOmega _0$ which in turn can be done in two steps. First, we compute the convex hull of the union of the region $\mathcal {R}$ and its image at the moment $T_\delta$. Second, we observe that the continuous dynamics non-linearities can lead to some reachable states being outside of the computed convex hull. In order to account for this phenomena, we bloat the resulting convex hull to ensure the over-approximation. Clearly, a larger sampling time $T_\delta$ makes a possibly larger bloating necessary, which worsens the approximation precision (see Figs. 4 and 5 for a comparison).

To summarize, we observe that the adjustment of the template directions used in the support function representation and the sampling time in the continuous post operator crucially impacts the precision, i.e., the *abstraction level*, of the symbolic state representation. Clearly, an improved precision leads to an increased analysis time on the downside. Based on this representation, we will present an algorithm which leverages different abstraction levels to efficiently explore the region space.

### Guided search

In this section, we introduce a guided search algorithm (Algorithm 1) along the lines of the reachability algorithm used by the current version of SpaceEx [23]. It works on the region space of a given hybrid system. The algorithm checks if a symbolic error state is reachable from a given set of initial symbolic states $\mathcal {S}_{ init }$. As outlined above, we define a symbolic state $s_e$ in the region space of $\mathcal {H}$ to be a symbolic error state if there is a symbolic state $s\in \mathcal {S}_{ bad }$ such that *s* and $s_e$ agree on their discrete part, and the intersection of the regions of *s* and $s_e$ is not empty (in other words, the error states are defined with respect to the given set of bad states). Starting with the set of initial symbolic states from $\mathcal {S}_{ init }$, the algorithm explores the region space of a given hybrid system by iteratively computing symbolic successor states until an error state is found, no more states remain to be considered, or a (given) maximum number of iterations $i_{ max }$ is reached. The exploration of the region space is guided by the $cost$ function such that symbolic states with lower cost values are considered first.
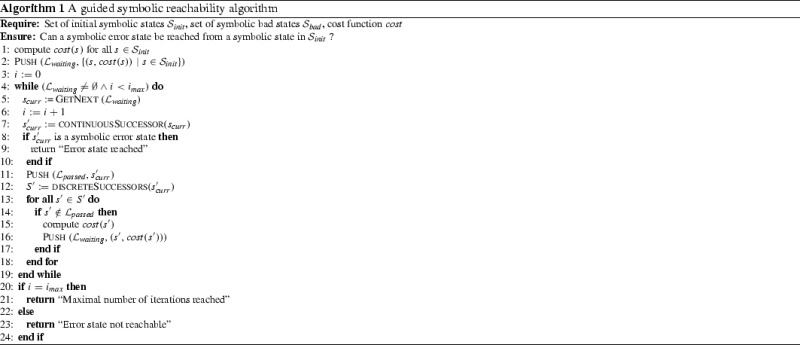


In the following, we provide a conceptual description of the algorithm using the following terminology. A symbolic state $s'$ is called a symbolic *successor state* of a symbolic state *s* if $s'$ is obtained from *s* by first computing the continuous successor of *s* (according to iteratively over-approximating the successor regions of *s* with sets $\varOmega _i$ as described in the previous section), and then by computing a discrete successor state of the resulting (intermediate) state. Therefore, for a given symbolic state $s_{ curr }$, the function continuousSuccessor (line 7) returns a symbolic state which is an over-approximation of the symbolic state reachable from $s_{ curr }$ within the given time horizon according to the continuous evolution. Accordingly, the function discreteSuccessors (line 12) returns the symbolic states that are reachable due to the outgoing discrete transitions.

A symbolic state *s* is called *explored* if its symbolic successor states have been computed. A symbolic state *s* is called *visited* if *s* has been computed but not yet necessarily explored. To handle encountered states, the algorithm maintains the data structures $\mathcal {L}_{ passed }$ and $\mathcal {L}_{ waiting }$. $\mathcal {L}_{ passed }$ is a list containing symbolic states that are already explored; this list is used to avoid exploring cycles in the region space. $\mathcal {L}_{ waiting }$ is a priority queue that contains visited symbolic states together with their cost values that are candidates to be explored next. The algorithm is initialized by computing the cost values for the initial symbolic states and pushing them accordingly into $\mathcal {L}_{ waiting }$ (lines 1 – 2). The main loop iteratively considers a best symbolic state $s_{ curr }$ from $\mathcal {L}_{ waiting }$ according to the cost function (line 5), computes its symbolic continuous successor state $s_{ curr }'$ (line 7), and checks if $s_{ curr }'$ is a symbolic error state (lines 8 – 10). (Recall that $s_{ curr }'$ is defined as a symbolic error state if there is a symbolic bad state $s\in \mathcal {S}_{ bad }$ such that *s* and $s_{ curr }'$ agree on their discrete part, and the intersection of the regions of *s* and $s_{ curr }'$ is not empty.) If this is the case, the algorithm terminates. If this is not the case, then $s_{ curr }'$ is pushed into $\mathcal {L}_{ passed }$ (line 11). Finally, for the resulting symbolic state $s_{ curr }'$, the symbolic discrete successor states are computed, prioritized, and pushed into $\mathcal {L}_{ waiting }$ if they have not been considered before (lines 12 – 18). As a side remark, if a successor state $s'=\langle l,\mathcal R\rangle$ is not contained in $\mathcal {L}_{ passed }$ (line 14), but instead there is a symbolic state $s''=\langle l,\mathcal R'\rangle \in \mathcal {L}_{ passed }$ with $\mathcal R\subset \mathcal R'$, then $s'$ is discarded as well because all transitions enabled in $s'$ have already been enabled in $s''$ which is already explored. Finally, the check if the given maximal number of iterations has been reached (line 4 and line 20) ensures termination, which would not be generally guaranteed otherwise (e. g., because of Zeno behavior).

Obviously, the search behavior of Algorithm 1 is crucially determined by the cost function that is applied. In the next section, we give a generic description of *pattern database* cost functions.

### General framework of pattern databases

For a given system $\mathcal {S}$, a pattern database (PDB) in the classical sense (i. e., in the sense PDBs have been considered for discrete and timed systems) is represented as a table-like data structure that contains abstract states together with abstract cost values. The PDB is used as a cost estimation function by mapping concrete states *s* to corresponding abstract states $s^{\#}$ in the PDB, and using the abstract cost value of $s^{\#}$ as an estimation of the cost value of *s*. The computation of a classical PDB is performed in three steps. First, a subset $\mathcal {P}$ of variables and automata of the original system $\mathcal {S}$ is selected. Such subsets $\mathcal {P}$ are called *pattern*. Second, based on $\mathcal {P}$, an abstraction $\mathcal {S}^{\#}$ is computed that only keeps the variables occurring in $\mathcal {P}$. Third, the entire state space of $\mathcal {S}^{\#}$ is computed and stored in the PDB. More precisely, all reachable abstract states together with their abstract cost values are enumerated and stored. The abstract cost value for an abstract state is defined as the shortest length of a trajectory from that state to an abstract error state. The resulting PDB of these three steps is used as the $cost$ function during the execution of Algorithm 1; in other words, the PDB is computed *prior* to the actual model checking process, where the resulting PDB is used as an input for Algorithm 1.

A straight-forward adaptation of such classical PDBs to the area of hybrid systems is the following. For a given hybrid system $\mathcal {H}$, compute an abstract system $\mathcal {H}^{\#}$ as the basis for the PDB, where $\mathcal {H}^{\#}$ is obtained from $\mathcal {H}$ by removing some of the variables in $\mathcal {H}$ (the pattern corresponds to the remaining variables in $\mathcal {H}^{\#}$). Based on $\mathcal {H}^{\#}$, the PDB is represented by a data structure that contains abstract states together with corresponding cost values. The abstract states and cost values are obtained by a region space exploration of $\mathcal {H}^{\#}$. The abstract cost value of an abstract state $s^{\#}$ is defined as the length of a shortest found trajectory in $\mathcal {H}^{\#}$ from $s^{\#}$ to an abstract error state. The PDB computes the cost function

$$\begin{aligned} cost ^{ P }(s):= cost ^{\#}(s^{\#}), \end{aligned}$$

where *s* is a symbolic state, $s^{\#}$ is a corresponding abstract state to *s* in the PDB, and $cost ^{\#}$ is the length of the corresponding trajectory from $s^{\#}$ to an abstract error state as defined above.

## Pattern databases for hybrid systems

In Sect. 2.4, we have described the general approach for computing and using a PDB for guiding the search. However, for hybrid systems, there are several challenges using the classical PDB approach. First, it is not clear how to effectively design and compute suitable abstractions $\mathcal {H}^{\#}$ for hybrid systems $\mathcal {H}$ with complex variable dependencies. Second, in Sect. 3.2, we address the general problem that the precomputation of a PDB is often quite expensive, where in many cases, only a small fraction of the PDB is actually needed for the search [24]. This is undesirable in general, and specifically becomes problematic in the context of hybrid systems because the reachability analysis in hybrid systems is typically much more expensive than, e. g., for discrete systems. In Sect. 3.2, we introduce a variant of *partial* PDBs for hybrid systems to address these problems.

### Coarse-grained space abstractions

A general question in the context of PDBs is how to compute suitable abstractions of a given system. In particular, for hybrid systems where variables often have rather complex dependencies, projection abstractions based on removing variables (as done for classical PDBs) can be too coarse to achieve accurate heuristics. In this paper, we propose a simple, yet elegant alternative to the classical PDB approach to obtain a coarse grained and fast analysis: As described in Sect. 2.2, the LeGuernic-Girard (LGG) algorithm implemented in SpaceEx [23] uses support function representation (based on the chosen set of template directions) to compute and store over-approximations of the reachable states. Therefore, a reduced number of *template directions* and an increased *sampling time* results in an abstraction of the original region space in the sense that the dependency graph of the reachable abstract symbolic states is a discrete abstraction of the system.

The granularity of the resulting abstraction is directly correlated with the parameter selection: Choosing coarser parameters (fewer template directions, larger sampling time) in the reachability algorithm makes this abstraction coarser, whereas finer parameters lead to finer abstractions as well. In more detail, for a given set of template directions *D* and sampling time *N*, a subset $D'\subset D$ and a larger sampling time $N'> N$ induce *coarse-grained space abstractions* with respect to the abstractions obtained by *D* and *N*: the over-approximation of regions based on $D'$ and $N'$ are coarser than for *D* and *N*. As an example for template directions, consider again Figs. 2 and 3: the set of box directions in Fig. 2 is a coarse-grained space abstraction of the set of octagonal directions in Fig. 3. Similarly, as an example for the sampling time, consider again Figs. 4 and 5, where Fig. 4 shows a coarse-grained space abstraction based on increased sampling time of the regions in Fig. 5.

In the following, we apply coarse-grained space abstractions to obtain abstractions as the basis for pattern databases. This is a significant difference compared to classical PDB approaches (see Sect. 2.4): Instead of computing an explicit (projection) abstraction $\mathcal {H}^{\#}$ based on a *subset* of all variables, we *keep* all variables (and hence, the original system $\mathcal {H}$), and instead choose a coarser exploration of the abstract region space of $\mathcal {H}$ to obtain the abstraction used for the PDB. (In practice, we apply unguided search provided by SpaceEx to compute this coarser abstraction.) As an additional difference to classical PDBs, we will apply a variant of *partial* PDBs, which are introduced in the next section.

### Partial pattern databases

As already outlined, a general drawback of classical PDBs is the fact that their precomputation might become quite expensive. Even worse, in many cases, most of this precomputation time is often unnecessary because only a small fraction of the PDB is actually needed during the symbolic search in the region space [24]. One way that has been proposed in the literature to overcome this problem is to compute the PDB on demand: The so-called *switchback search* maintains a family of abstractions with increasing granularity; these abstractions are used to compute the PDB to guide the search in the next finer level [32].

In the following, we apply a variant of *partial* PDBs [7] based on coarse-grained space abstractions to address this problem: Instead of computing the whole abstract region space for a given abstraction, we restrict the abstract search to explore only a fraction of the abstract region space while focusing on those abstract states that are likely to be sufficient for the concrete search. In the following definition, we call an abstract state $s^{\#}$*corresponding* to state *s* if *s* and $s^{\#}$ agree on their discrete part, the region of *s* is included in region of $s^{\#}$, and $s^{\#}$ is an abstract state with minimal abstract costs that satisfies these requirements.

#### **Definition 2**

(*Partial Pattern Database*) Let $\mathcal {H}$ be a hybrid system. A *partial pattern database* for $\mathcal {H}$ is a pattern database for $\mathcal {H}$ that contains only abstract state/cost value pairs for abstract states that are part of some trajectory of shortest length (in terms of number of location switches) from an initial state to some abstract error state. The partial pattern database computes the function

$$\begin{aligned} cost ^{ PP }(s):= \left\{ \begin{array}{l l} cost ^{\#}(s^{\#}) &{} \quad \text {if ex. corresponding } s^{\#}\text { to } s\\ +\infty &{} \quad \text {otherwise}\\ \end{array} \right. \end{aligned}$$

where *s*, $s^{\#}$, and $cost ^{\#}$ are defined as above, and +$\infty$ is a default value indicating that no corresponding abstract state to *s* exists.

Informally, a partial PDB for a hybrid system $\mathcal {H}$ exactly contains those abstract states that are explored on some *shortest* trajectory (instead of containing *all* abstract states of a complete abstract region space exploration to *all* abstract error states as it would be the case for a classical PDB). In other words, partial PDBs are incomplete in the sense that there might exist concrete states without any corresponding abstract states in the PDB. In such cases, the default value +$\infty$ is returned with the intention that corresponding concrete states are only explored if no other states are available. Obviously, this might worsen the overall search guidance compared to the fully computed PDB. However, in special cases, a partial PDB is already sufficient to obtain the same cost function as obtained with the original PDB or even obtained with a perfect cost function (that allows for exploring the region space without backtracking to find an error state). For example, this is the case when only abstract states are excluded from which no abstract error state is reachable anyway. More generally, under the idealized assumption that the abstraction is fine enough such that no spurious behavior occurs on shortest possible error trajectories, the partial PDB already delivers the same search behavior as a perfect search algorithm that finds an error trajectory without backtracking.

#### **Proposition 1**

Let $$\mathcal {H}$$ be a hybrid system. Let $$n\in \mathbb {N}_0$$ be the length of a shortest concrete error trajectory. If all shortest abstract error trajectories in $$\mathcal {H}$$ (obtained by a coarse-grained space abstraction to build a pattern database) correspond to concrete error trajectories of the same length, then guided search with Algorithm 1 and $$ cost ^{ PP }$$ finds an error trajectory after *n* steps.

#### *Proof*

By construction, the partial PDB contains exactly those symbolic abstract states that are part of shortest possible error trajectories. By assumption, these abstract states correspond to concrete states on concrete error trajectories of the same length. Hence, for every concrete state *s* on a shortest error trajectory, there is a corresponding entry in the partial PDB for all concrete successor states $$s'$$ of *s* that are part of a shortest concrete error trajectory, and $$ cost ^{ PP }(s')= cost ^{ PP }(s)-1$$. In addition, for all concrete successor states $$s''$$ that are not part of a shortest concrete error trajectory, $$ cost ^{ PP }(s'')=\infty $$. Overall, the claim follows by an inductive argument: Let $$s_0$$ be an initial state such that $$ cost ^{ PP }(s_0)=n$$ is minimal among the costs of all initial states, i. e., *n* is the length of a shortest concrete error trajectory. Furthermore, all concrete states on a shortest concrete error trajectory have a concrete successor state with a cost value decreased by one, whereas all other successor states have a cost value of infinity. Hence, Algorithm 1 with the $$ cost ^{ PP }$$ function finds a concrete error trajectory within *n* steps. $$\square $$

Under the idealized assumptions of Proposition 1, it follows immediately that guided search applying the full PDB cannot improve over the partial PDB.

#### **Corollary 1**

Under the assumptions of Proposition 1, guided search with Algorithm 1 and $$ cost ^{ P }$$ explores at least as many states as with $$ cost ^{ PP }$$.

Proposition 1 and Corollary 1 show that partial PDBs can provide effective search guidance in an idealized setting where the applied abstraction only introduces spurious behavior on non-relevant parts of the region space. Clearly, in practice, these assumptions will mostly not be satisfied for abstractions that are efficiently computable. However, we rather consider Proposition 1 as a proof of concept showing that the basic concept of partial PDBs is meaningful in our setting. (In our experimental analysis, we will show that partial PDBs yield an effective and efficient approach for a number of practical and challenging problems as well—we will come back to this point in Sect. 5.) Overall, we will see that although in case the requirements of Proposition 1 are not fulfilled, partial PDBs can still be a good heuristic choice that lead to cost functions that are efficiently computable and accurately guide the concrete search.

### Discussion

Our pattern database approach for finding error states exploits abstractions in a different way than in common approaches for verification (see Sect. 4 for a discussion on related work). Most notably, the main focus of our abstraction is to provide the basis for the cost function to guide the search, rather than to prove correctness (although, under certain circumstances, it can be efficiently used for verification as well—we will come back to this point in the experiments section). As a short summary of the overall approach, we first compute a symbolic abstract region space (as described in Sect. 3.1), where the encountered symbolic abstract states $s^{\#}$ are stored in a table together with the corresponding abstract cost values of $s^{\#}$. To avoid the (possibly costly) computation of an *entire* PDB, we only compute the PDB partially (as described in Sect. 3.2). This partial PDB is then used as the cost function of our guided reachability algorithm. As in many other approaches that apply abstraction techniques to reason about hybrid systems, the abstraction that is used for the PDB is supposed to accurately reflect the “important” behavior of the system, which results in accurate search guidance of the resulting cost function and hence, of our guided reachability algorithm.

An essential feature of the PDB-based cost function is the ability to reflect the continuous *and* the discrete part of the system. To make this more clear, consider again the motivating example from the introduction (Fig. 1). As we have discussed already, the box-based distance function first wrongly explores the whole lower branch of this system because no discrete information is used to guide the search. In contrast, a partial PDB is also able to reflect the discrete behavior of the system. In this example, the partial PDB consists of an abstract trajectory to the first reachable error state, which is already sufficient to guide the (concrete) region space exploration towards to first reachable error state as well. In particular, this example shows the advantage of partial PDBs compared to fully computed PDBs (recall that fully computed PDBs would include *all* error states, whereas the partial PDB only contains the trajectory to a shortest one). In general, our PDB approach is particularly well suited for hybrid systems with a non-trivial amount of discrete behavior. However, the continuous behavior is still considered according to our abstraction technique as introduced in Sect. 3.1. Overall, partial PDBs appear to be an accurate approach for guided search because they accurately balance the computation time for the cost function on the one hand, and lead to efficient and still accurately informed cost functions on the other hand.

## Related work

Abstraction techniques for hybrid systems have been mostly considered in a verification setting, i. e., in a setting where the focus is on proving that a given set of bad states cannot be reached. For this purpose, abstractions have been applied in different ways. On the one hand, a number of approaches to abstract the *regions* of symbolic states within the reachability analysis have been suggested, including constraint polyhedra [22], ellipsoids [31], and orthogonal polyhedra [15]. In our paper, we use the support function representation [33]. These approaches have in common that the structure of the considered hybrid system is left intact. On the other hand, it also possible to abstract a hybrid automata structure. Alur et al. [2] suggest to use predicate abstraction for the hybrid systems analysis. In addition, Tiwari et al. [40] introduce a method based on the quantifier elimination decision procedure for real closed fields. Furthermore, Tiwari [39] investigates Lie derivatives and their application to the abstraction generation. Jha et al. [25] computes abstractions by removing some of the continuous variables. Finally, Bogomolov et al. [13] abstract hybrid systems by merging locations. The abstract dynamics is computed by eliminating the state variables and computing a convex hull. Our pattern database approach belongs to the first group outlined above as we exploit the parametrization of the symbolic region representation.

A prominent model checking approach for hybrid systems is based on counterexample-guided abstraction refinement (CEGAR) [3, 4]. In a nutshell, CEGAR iteratively refines the considered abstraction until the abstraction is fine enough to prove or refute the property. Our PDB approach shares with CEGAR the general idea of using an abstraction to analyze a concrete system. However, in contrast to CEGAR, where abstract counterexamples have to be validated and possibly used in further abstraction refinement, abstractions for PDBs are never refined and only used as a heuristic to *guide* the search within the concrete automaton. In other words, in contrast to CEGAR, the accuracy of the abstraction influences the *order* in which concrete states are explored, and hence, the accuracy in turn influences the *performance* of the resulting model checking algorithm.

Therefore, a crucial difference lies in the fact that CEGAR does the search in the abstract space, replays the counterexample in the concrete space, and stops if the error trajectory cannot be followed. In contrast, our approach does the search in the concrete space and uses the PDBs for guidance, only. If an abstract trajectory cannot be followed, the search does not stop, but tries other branches until either a counterexample is found, or all trajectories have been exhausted. Due to this reason, our framework provides the same level of precision as the default SpaceEx reachability algorithm. The concretization of a symbolic path is known to be a highly non-trivial computational problem. A symbolic bad path found with our approach can be further concretized to the trajectory level using techniques from optimal control (see, e.g., the work by Zutshi et al. [43] for more details).

Considering more specialized techniques to find error states in faulty hybrid systems, Bhatia and Frazzoli [11] propose using rapidly exploring random trees (RRTs). In the context of hybrid systems, the objective of a basic RRTs approach is to efficiently cover the region space in an “equidistant” way in order to avoid getting stuck in some part of the region space. Recently, RRTs were extended by adding guidance of the input stimulus generation [18]. However, in contrast to our approach, RRTs approaches are based on numeric simulations, rather than symbolic executions. Applying PDBs to RRTs would be an interesting direction for future work. In a further approach, Plaku, Kavraki, and Vardi [36] propose to combine motion planning with discrete search for falsification of hybrid systems. The discrete search and continuous search components are intertwined in such a way that the discrete search extracts a high-level plan that is then used to guide the motion planning component. In a slightly different setting, Ratschan and Smaus [38] apply search to finding error states in hybrid systems that are deterministic. Hence, the search reduces to the problem of finding an accurate initial state.

SpaceEx [23] is a recently developed, yet already prominent model checking tool for hybrid systems. As suggested by the name, it explores the region space by applying (symbolic) search. The most related approach to this paper has recently been presented by Bogomolov et al. [14], who propose a cost function based on Euclidean distances of the regions of the current state and error states. The resulting guided search algorithm is implemented in SpaceEx and has demonstrated to achieve significant guidance and performance improvements compared to the uninformed search of SpaceEx. In contrast to the presented PDB approach of this paper, the Euclidean distances are solely based on the continuous part of the system, whereas PDBs are able to reflect both discrete and continuous parts.

**Table 1:** Results for the navigation benchmarks

Inst.	#Loc	Uninformed DFS			Box-heuristic			PDB		
		#It	Length	Time	#It	Length	Time	#It	Length	Time (time abs.)
1	400	122	15	206.1	62	15	99.883	16	15	**28.325** (2.714)
2	400	183	33	262.565	86	33	168.815	34	33	**75.626** (10.153)
3	625	75	33	99.758	34	33	**52**.**222**	34	33	62.283 (10.234)
4	625	268	158	368.545	231	158	296.89	159	158	**178.705** (13.992)
5	625	85	79	167.502	26	25	**53**.**164**	26	25	58.417 (5.002)
6	625	96	53	155.458	101	53	148.448	54	53	**106.283** (13.267)
7	625	227	34	280.406	105	34	137.363	35	34	**66.315** (12.682)
8	625	178	25	371.8	86	25	192.71	26	25	**60.639** (9.609)
9	625	297	17	502.049	102	17	187.003	18	17	**42.785** (10.232)
10	625	440	30	753.488	136	30	282.914	31	30	**84.031** (18.114)
11	900	234	72	378.906	129	21	208.789	22	21	**45.085** (10.973)
12	900	317	43	473.785	174	61	277.467	44	43	**86.936** (21.097)
13	900	367	37	596.671	148	37	266.718	38	37	**97.456** (26.926)
14	900	411	32	608.962	278	32	419.827	33	32	**79.987** (14.934)
15	900	379	44	625.685	107	44	194.535	45	44	**97.138** (12.302)

Bold values indicate the best analysis method for every benchmark

*Uninformed DFS* uninformed depth-first search, *Box-heuristic* box-based distance heuristic, *PDB* our PDB cost function $$ cost ^{ PP }$$, *#loc* number of locations, *#it* number of iterations, *length* length of the found error trajectory, *time* total time in seconds including any preprocessing. For our PDB approach, the fraction of the total time that is needed for the PDB computation is additionally reported in parenthesis

Moreover, guided search has been applied to finding error states in a subclass of hybrid systems, namely to *timed* systems. In particular, PDBs have been investigated in this context [29, 30, 42]. In contrast to this paper, the PDB approaches for timed systems are “classical” PDB approaches, i. e., a subset of the available automata and variables are selected to compute a projection abstraction. To select this subset, Kupferschmid et al. [29] compute an abstract error trace and select the automata and variables that occur in transitions in this abstract trace. In contrast, Kupferschmid and Wehrle [30, 42] start with the set of all automata and variables (i. e., with the complete system), and iteratively remove variables as long as the resulting projection abstraction is “precise enough” according to a certain quality measure. In both approaches, the entire PDB is computed, which is more expensive than the partial PDB approach proposed in this paper.

## Evaluation

We have implemented $$ cost ^{ PP }$$ in the SpaceEx tool [23] and evaluated it on a number of challenging benchmarks. The implementation and the benchmarks are available at http://pub.ist.ac.at/~sbogomol/sttt2015.

The experiments have been performed on a machine running with AMD Opteron 6174 processors. We set a time limit of 30 min per run. In the following, we report results for our PDB implementation of $$ cost ^{ PP }$$ in SpaceEx. We compared $$ cost ^{ PP }$$ with uninformed DFS as implemented in SpaceEx, and with the recently proposed box-based distance function [14] on several challenging benchmark problems. We compare the number of iterations of SpaceEx, the length of the error trajectory found as well as the overall search time (including the computation of the PDB for $$ cost ^{ PP }$$) in seconds. In the following, we will shortly denote partial PDBs with PDBs.

### Results for navigation benchmarks

As a first set of benchmarks, we consider a variant of the well-known navigation benchmark [21]. This benchmark models an object moving on the plane which is divided into a grid of cells. The dynamics of the object’s planar position in each cell is governed by the differential equations $\dot{x}=v$, $\dot{v}=A(v-v_d)$, where $v_d$ stands for the targeted velocity in this location. Compared to the originally proposed navigation benchmark problem, we address a slightly more complex version with the following additional constraints. First, we add inputs allowing perturbation of object coordinates, i. e., the system of differential equations is extended to: $\dot{x}=v+u$, $\dot{v}=A(v-v_d)$, $u_{min} \le u \le u_{max}$. Second, to make the search task even harder, the benchmark problems also feature obstacles between certain grid elements. This is particularly challenging because, in contrast to the original benchmark system, one can get stuck in a cell where no further transitions can be taken, and consequently, backtracking might become necessary. The size of the problem instances varies from 400 to 900 locations, and all instances feature 4 variables.

**Fig. 6:**
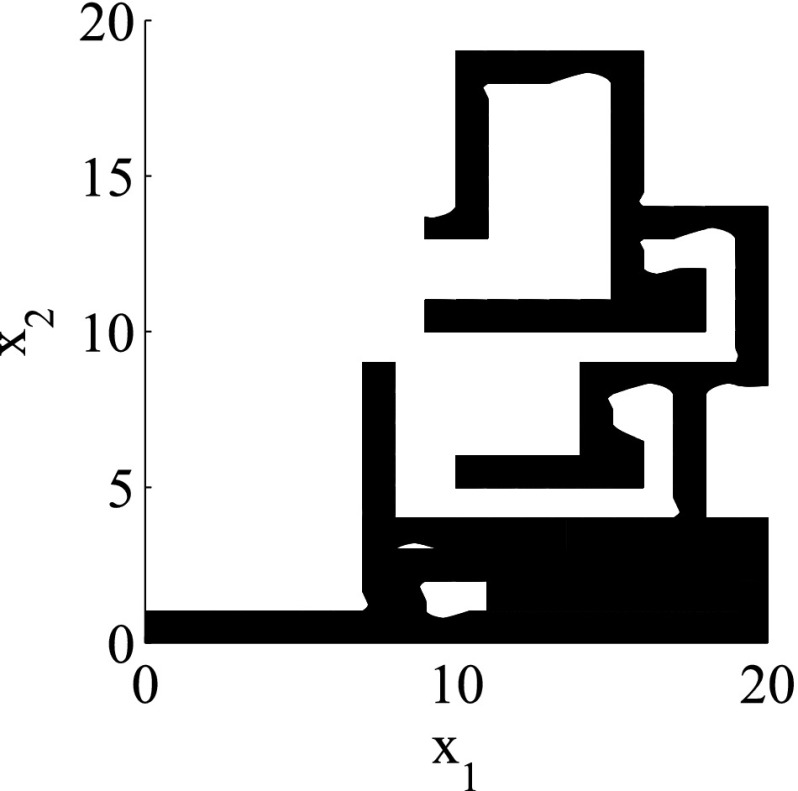
*Navigation benchmark* uninformed search error trajectory for instance 1

**Fig. 7:**
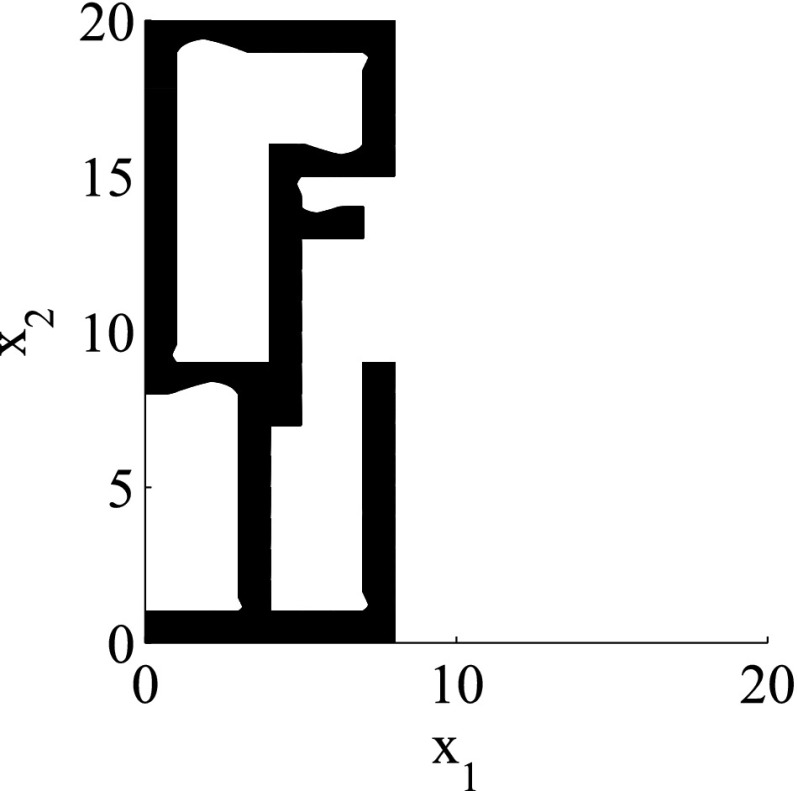
*Navigation benchmark* box-based heuristic search error trajectory for instance 1

**Fig. 8:**
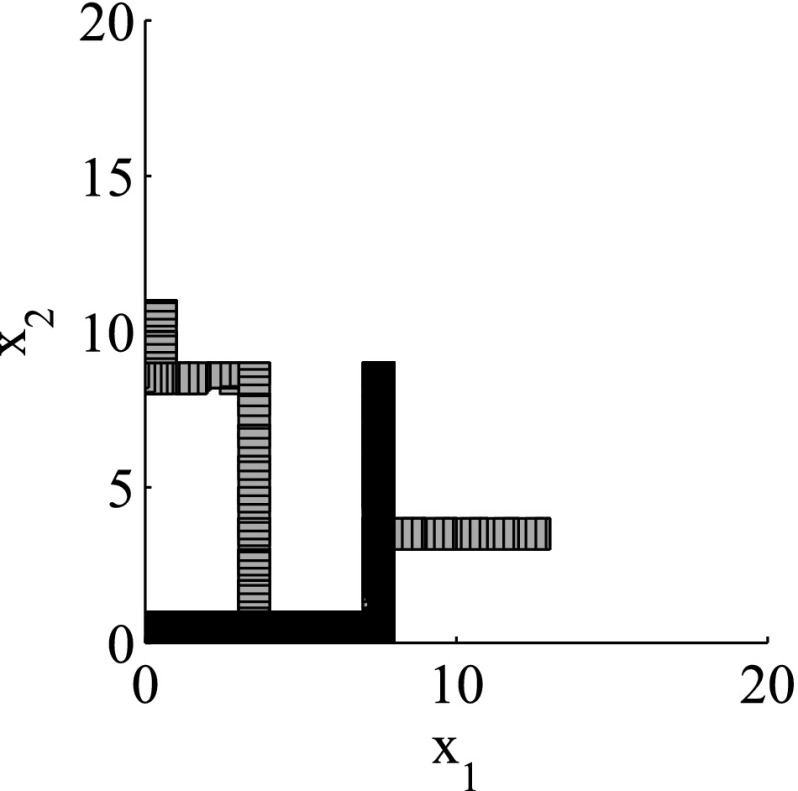
*Navigation benchmark* PDB search error trajectories for instance 1 (abstract: *light gray*, concrete: *dark gray*)

The results for the navigation benchmark problem class are provided in Table 1, where the best results are given in bold fonts with respect to the total runtime. The fraction of the total time to compute the PDB is given in parenthesis. As a general picture, they show that the precomputation time for the PDB mostly pays off in terms of guidance accuracy and overall runtime. Specifically, the overall runtime could (sometimes significantly) be reduced compared to uninformed search and also compared to the box-based heuristic. For example, in navigation instance 1, the precomputation for the PDB only needs around 3 s, leading to an overall runtime of around 28 s, compared to around 99 s with the box-based heuristic, and about 206 s with uninformed search. This search behavior for instance 1 is also visualized in Figs. 6, 7, and 8, showing the trajectories (i. e., the parts of the covered region space) with the different search approaches. We observe the following: While uninformed DFS explores quite a large number of unnecessary trajectories, the box-based heuristic already guides the search more accurately and finds an error state with much fewer detours. Considering the PDB approach, we observe that PDBs can guide the search even more accurately in the sense that no detours are explored at all, and hence, no backtracking is needed either. Furthermore, the covered parts of the region space is again much lower than both with uninformed search and the box-based heuristic. In addition, we observe that even the abstract run (shown in light gray) is already rather accurate, covering only little more of the region space than the concrete run. Overall, the PDB approach finds an accurate balance between the computation time and the accuracy of the resulting cost function.

#### Results for navigation benchmarks with additive vanishing perturbation

We consider another variant of the navigation benchmark with an additive vanishing perturbation (see, e.g., [28]). We use this variation for evaluating the scalability of our approach with respect to increased continuous complexity given a constant discrete complexity. For this, the benchmark is modified to model a vanishing perturbation $w \in \mathbb {R}^p$ with increasing model order (i. e., $p=1$, $p=2$, etc.). In more detail, we extend the system of differential equations to $\dot{x}=v+u$, $\dot{v}=A(v-v_d)+\sum _{i=1}^p w_i$, $\dot{w} = A_w w$, where $u_{min} \le u \le u_{max}$ as before and $A_w \in \mathbb {R}^{p \times p}$ is Hurwitz to ensure it is a vanishing perturbation.

**Fig. 9:**
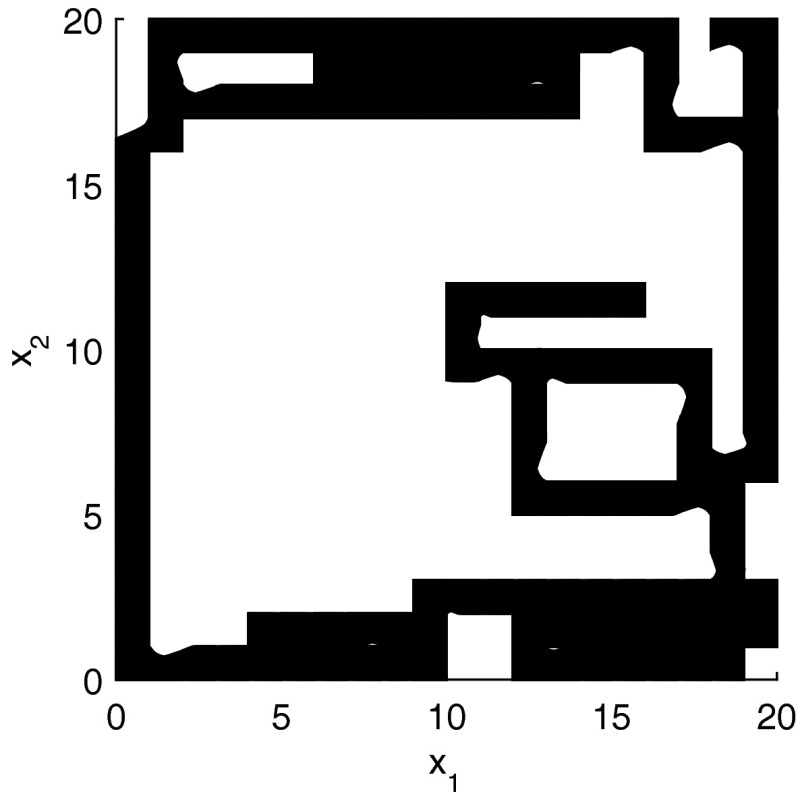
Navigation benchmark with additive vanishing perturbation for $p = 2$: uninformed search error trajectory for instance 2

**Fig. 10:**
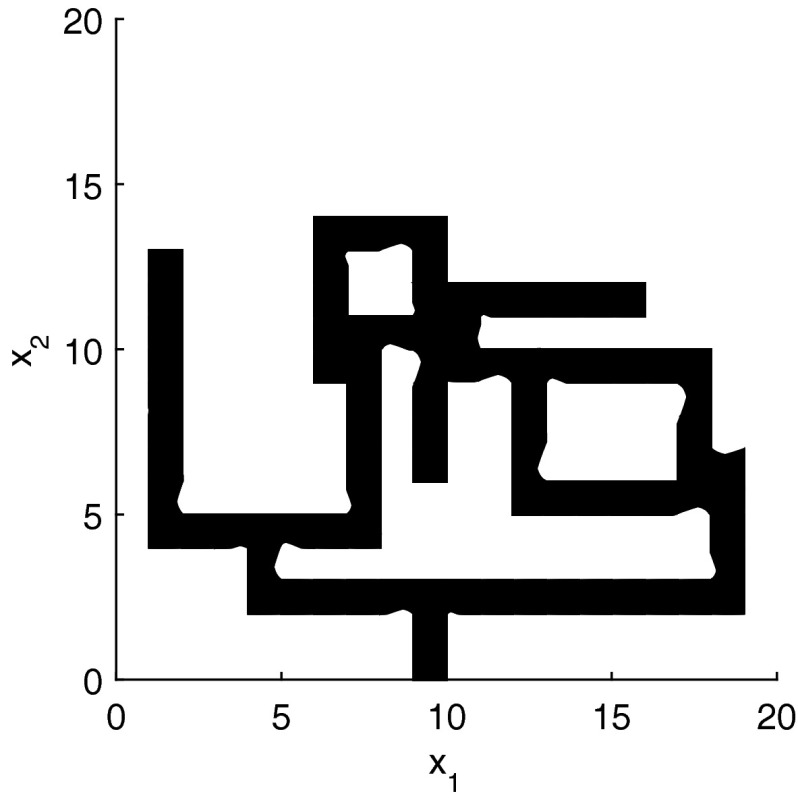
Navigation benchmark with additive vanishing perturbation for $p = 2$: box-based heuristic search error trajectory for instance 2

**Fig. 11:**
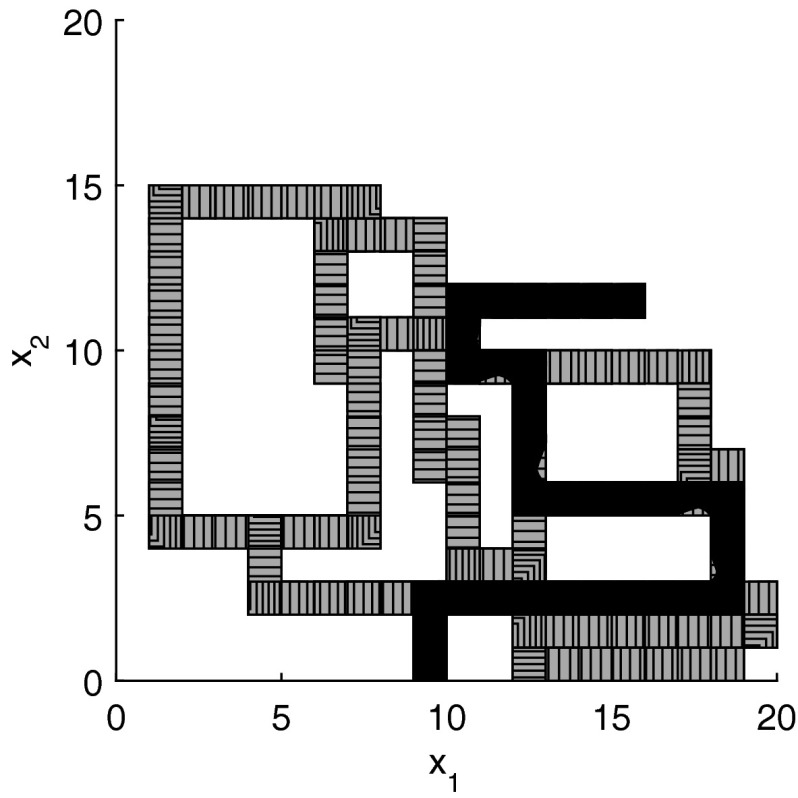
Navigation benchmark with additive vanishing perturbation for $p\,=\,2$: PDB search error trajectories for instance 2 (abstract: *light gray*, concrete: *dark gray*)

Table 2 presents results of the same scenarios evaluated in the earlier navigation benchmark for $p=2$ additional vanishing perturbation variables (i. e., 2 additional state variables compared to the earlier navigation benchmark, yielding $n=6$ continuous variables overall). We observe a similar picture as for the previous results: the PDB approach outperforms uninformed DFS and also the box-based heuristic in the majority of the problems. This is also reflected in Figs. 9, 10, and 11, which, respectively, show the corresponding reachable states in the second instance for the three approaches, respectively.

**Fig. 12:**
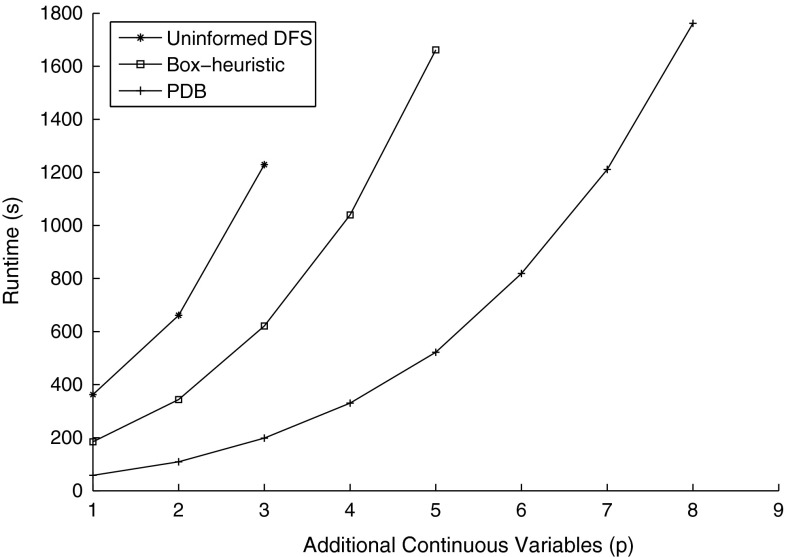
Navigation benchmark with additive vanishing perturbation for $1 \le p \le 8$ with the same discrete structure as instance 1 (i. e., all else constant except the number of additive perturbation terms). The total number of continuous variables is $n=4+p$

In addition, Fig. 12 presents the navigation benchmark instance 1 scaling the number of additional state variables from $p=1$ through $p=8$ (for a total of $n = 5$ through $n = 12$ continuous variables), while keeping all else constant, using a timeout of 30 min, and runs that exceeded the 30 min timeout are not plotted. We observe that also with increasing number of additional continuous variables, the runtime scalability of our PDB approach is considerably better compared to the box-based heuristic and uninformed DFS—even for $p=8$ additional variables, PDBs is able to find an error state in less than 30 min. In contrast, both the uninformed DFS and box-based heuristic methods cannot find the error states in less than 30 min beyond $p=3$ and $p=5$ additional variables, respectively.

**Table 2:** Results for the navigation benchmarks with two additional continuous variables modeling an additive vanishing perturbation

Inst.	#Loc	Uninformed DFS			Box-heuristic			PDB		
		#It	Length	Time	#It	Length	Time	#It	Length	Time (time abs.)
1	400	122	15	661.373	62	15	343.611	16	15	**109.264** (4.467)
2	400	153	33	723.719	86	33	592.531	34	33	**254.383** (17.427)
3	625	75	33	349.476	34	33	**199**.**073**	34	33	216.528 (17.998)
4	625	268	158	1053.63	231	158	868.593	177	158	**627.206** (25.12)
5	625	85	79	525.811	26	25	**201**.**759**	26	25	211.146 (8.392)
6	625	96	53	500.29	101	53	485.165	54	53	**337.038** (23.205)
7	625	227	34	1040.75	105	34	535.871	35	34	**251.452** (24.724)
8	625	201	25	1311.04	88	25	697.649	26	25	**206.585** (15.822)
9	625	298	17	1640.59	102	17	658.379	18	17	**142.197** (17.881)
10	625	n/a	n/a	OOT	163	30	1212.59	31	30	**281.356** (31.616)
11	900	201	72	1013.24	129	21	710.073	22	21	**146.15** (19.058)
12	900	316	43	1530.46	174	61	928.734	44	43	**272.088** (35.26)
13	900	n/a	n/a	OOT	148	37	858.674	38	37	**293.259** (45.617)
14	900	n/a	n/a	OOT	278	32	1403.78	33	32	**260.377** (25.744)
15	900	n/a	n/a	OOT	163	52	988.228	100	52	**649.809** (20.265)

Bold values indicate the best analysis method for every benchmark

*OOT* out of time (max 30 min), *Uninformed DFS* uninformed depth-first search, *Box-heuristic* box-based distance heuristic, *PDB* our PDB cost function $$ cost ^{ PP }$$, *#loc* number of locations, *#it* number of iterations, *length* length of the found error trajectory, *time* total time in seconds including any preprocessing

### Results for satellite benchmarks

In this section, we consider benchmarks that result from *hybridization*. For a hybrid system $\mathcal {H}$ with non-linear continuous dynamics, hybridization is a technique for generating a hybridized hybrid automaton from $\mathcal {H}$. The hybridized automaton has simpler continuous dynamics (usually affine or rectangular) that over-approximate the behavior of $\mathcal {H}$ [8], and can be analyzed by SpaceEx. For our evaluation, we consider benchmarks from this hybridization technique applied to non-linear *satellite orbital dynamics* [27], where two satellites orbit the earth with non-linear dynamics described by Kepler’s laws. The orbits in three-dimensional space lie in a two-dimensional plane and may in general be any conic section, but we assume the orbits are periodic, and hence circular or elliptical. Fixing some orbital parameters (e.g., the orientations of the orbits in three-space), the states of the satellites in three-dimensional space $x_1, x_2 \in \mathbb {R}^3$ can be completely described in terms of their true anomalies (angular positions). Likewise, one can transform between the three-dimensional state description and the angular position state description. The non-linear dynamics for the angular position are $\dot{\nu }_i = \sqrt{\mu / p_i^3} (1 + e_i \cos \nu _i)^2$ for each satellite $i \in \{1, 2\}$, where $\mu$ is a gravitational parameter, $p_i = a_i (1 - e_i^2)$ is the semi-latus rectum of the ellipse, $a_i$ is the length of the semi-major axis of the ellipse, and $0 \le e_i < 1$ is the eccentricity of the ellipse (if $e_i = 0$, then the orbit is circular and $p_i$ simplifies to the radius of the circle). These dynamics are periodic with a period of $2\pi$, so we consider the bounded subset $[0,2\pi ]^2$ of the state-space $\mathbb {R}^2$, and add invariants and transitions to create a hybrid automaton ensuring $\nu _i \in [0, 2\pi ]$. For the benchmark cases evaluated, we fixed $\mu = 1$ and varied $p_i$ and $e_i$ for several scenarios. For more details, we refer to the work of Johnson et al. [27]. The size of the problem instances varies from 36 to 1296 locations, and all instances feature 4 variables.

**Table 3:** Results for the satellite benchmarks

Inst.	#Loc	Uninformed DFS			Box-heuristic			PDB		
		#It	Length	Time	#It	Length	Time	#It	Length	Time (time abs.)
1	36	116	32	37.501	75	10	18.393	16	10	**14.03** (10.05)
2	36	464	49	138.149	473	19	162.666	30	13	**21.882** (16.427)
3	64	719	87	42.198	281	91	**14.897**	264	121	27.067 (12.591)
4	100	111	106	51.705	45	15	30.602	23	14	**20.461** (8.106)
5	100	109	104	426.37	45	15	272.133	23	14	**92.393** (8.082)
6	159	2170	$\infty$	107.68	1354	$\infty$	68.107	0	$\infty$	**21.051** (21.051)
7	324	580	135	251.016	1289	144	649.345	25	24	**42.316** (11.921)
8	557	1637	42	61.754	936	42	**35.523**	156	42	60.027 (54.115)
9	574	7113	41	298.601	561	10	23.977	14	10	**8.811** (8.309)
10	575	9092	4	376.935	387	5	16.467	15	4	**3.182** (2.651)
11	576	1485	775	273.265	253	13	50.172	15	13	**13.385** (7.899)
12	576	1005	775	172.192	796	13	160.342	15	13	**13.324** (7.841)
13	576	1317	1147	1410.17	484	54	775.325	52	51	**217.534** (104.304)
14	1293	13691	$\infty$	526.483	7790	$\infty$	312.288	0	$\infty$	**170.428** (170.428)
15	1296	n/a	n/a	OOT	n/a	n/a	OOT	206	139	**784.986** (526.336)

Bold values indicate the best analysis method for every benchmark

*OOT* out of time (max 30 min), *Uninformed DFS* uninformed depth-first search, *Box-heuristic* box-based distance heuristic, *PDB* our PDB cost function $$ cost ^{ PP }$$, *#loc* number of locations, *#it* number of iterations, *length* length of the found error trajectory, *time* total time in seconds including any preprocessing

The verification problem is *conjunction avoidance*, i. e., to determine whether there exists a trajectory where the satellites come too close to one another and may collide. Some of the benchmark instances considered are particularly challenging because they feature several sources of non-determinism, including several initial states and several bad states. As an additional source of non-determinism, some benchmarks model thrusting. A change in a satellite’s orbit is usually accomplished by firing thrusters. This is usually modeled as an instantaneous change in the orbital parameters $e_i$ and $a_i$. However, the angular position $\nu _i$ in this new orbit does not, in general, equal the angular position in the original orbit, and a change of variables is necessary, which can be modeled by a reset of the $\nu _i$ values when the thrusters are fired. The transitions introduced for thrusting add additional discrete non-determinism to the system.

**Fig. 13:**
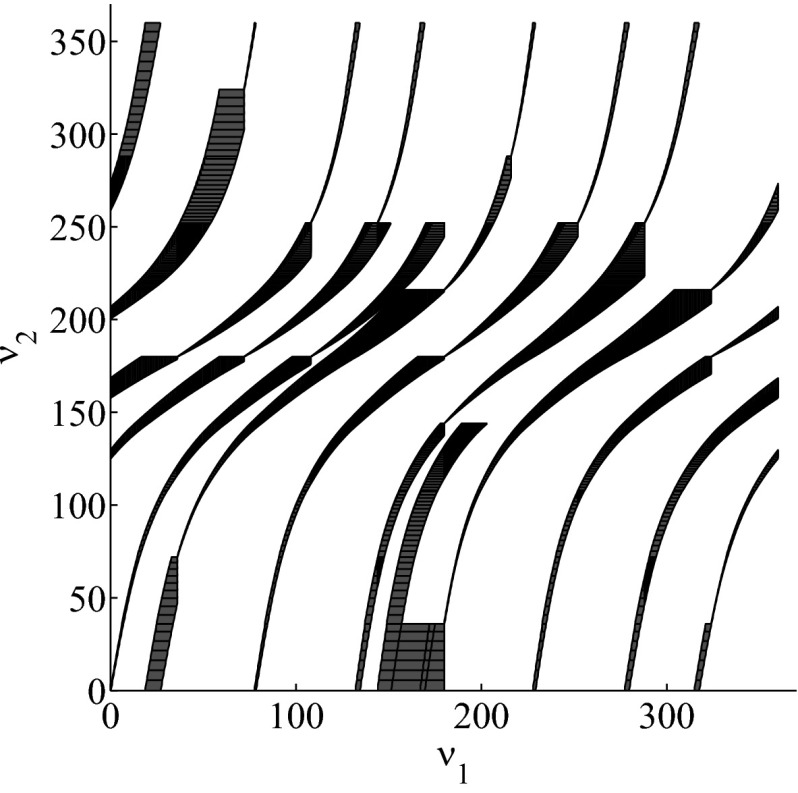
*Satellite benchmark*: uninformed search error trajectory for instance 5

**Fig. 14:**
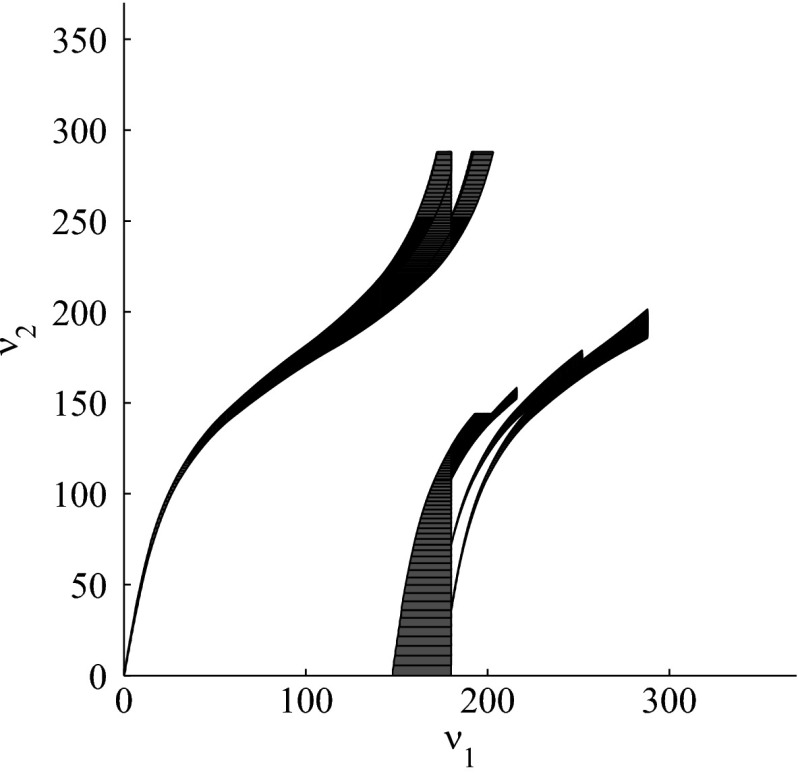
*Satellite benchmark*: box-based heuristic search error trajectory for instance 5

**Fig. 15:**
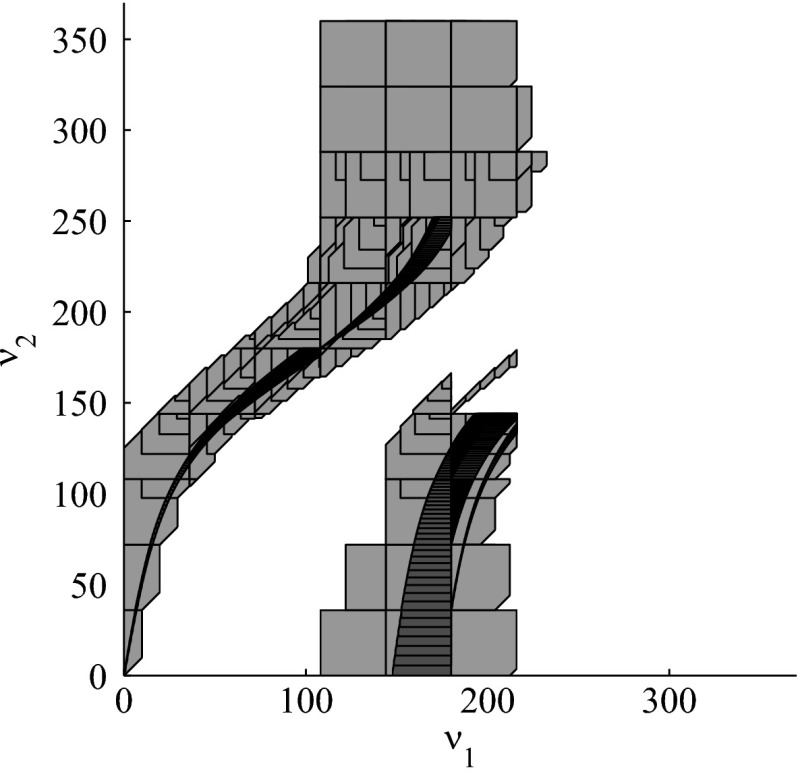
*Satellite benchmark*: PDB search error trajectories for instance 5 (abstract: *light gray*, concrete: *dark gray*)

The results for the satellite benchmark class are provided in Table 3. In general, we observe a similar search behavior to what we have observed in the navigation problems: The precomputation of the PDB pays off in the sense that much better search behavior can be achieved, leading to a fewer number of iterations and a lower overall runtime. For example, in instance 5, the precomputation time for the PDB amounts to roughly 5 s, leading to an overall time of around 92 s for the concrete run. In contrast, uninformed search and the box-based heuristic need around 426 and 272 s, respectively. The search behavior of the concrete and abstract run in instance 5 is also visualized in Figs. 13, 14, and 15. We observe that the part of the covered search space with our PDB approach is again lower compared to the box-based heuristic and uninformed search. Figure 15 again particularly shows the part of the search space that is covered by the abstract run (which can be performed efficiently due to our abstraction described in Sect. 3.1), showing that our PDB approach finds an accurate balance between the computation time and the accuracy of the resulting cost function.

Furthermore, we have also been able to effectively and efficiently prove the absence of errors in the instances 6 and 14, where the abstract run already revealed that no concrete error trajectory exists. As our abstraction is an over-approximation, we can safely conclude that no reachable error state in the concrete system exists either, and do not need to start the concrete search at all. Being able to efficiently verify hybrid systems with PDBs is a significant advantage compared to the box-based heuristic.

### Results for water tank benchmarks

**Fig. 16:**
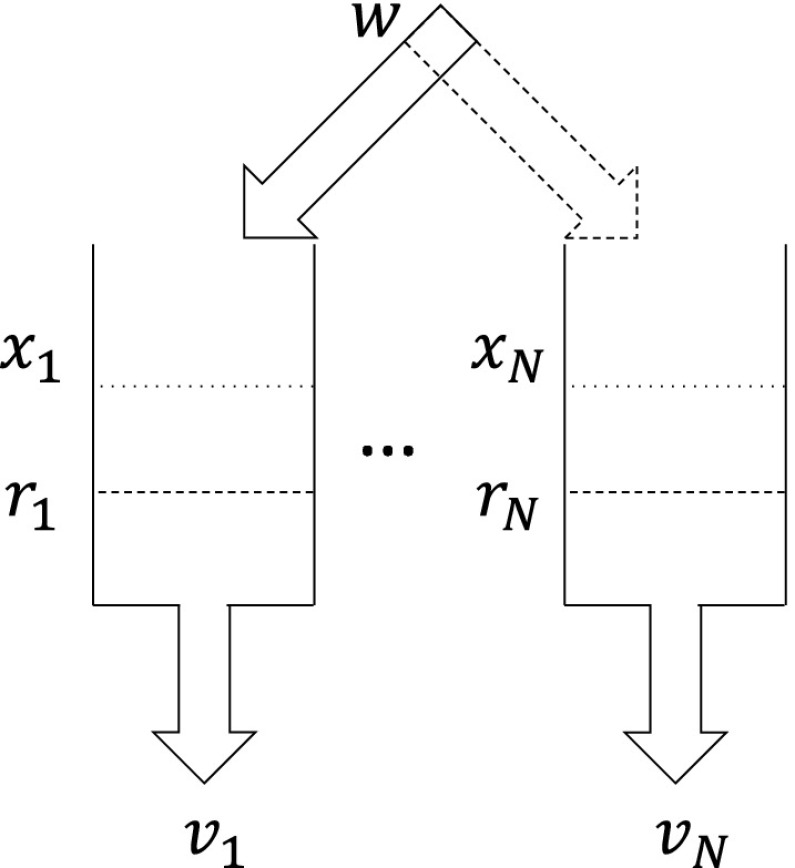
Water tank benchmark with *N* tanks

This benchmark consists of variants of the tank benchmark [6, 26]. The tank benchmark (see Fig. 16) consists of some $N \in \mathbb {N}$ tanks, where each tank $i \in \{1,\ldots ,N\}$ loses volume $x_i$ at some constant flow rate $v_i$, so tank *i* has dynamics $\dot{x}_i = -v_i$, for a real constant $v_i \ge 0$. One of the tanks is filled from an external inlet at some constant flow rate *w*, so it has dynamics $\dot{x}_i = w - v_i$, for a real constant $w \ge 0$. In our variant, the volume lost by each tank simply vanishes and does not move from one tank to another. This benchmark class is qualitatively different than either the navigation or satellite benchmarks, as the discrete state space may be small.

The two variations we consider are complete and linear topologies with regard to the inlet tank choice. The inlet pipe *wmay* be moved to some tank *j* with volume $x_i \le r_i$ from some tank *i*, where: (a) $j \ne i$ is any other tank for the complete topology, or (b) $j \in \{i+1, i-1\}$ is an adjacent tank for the linear topology. The invariants in our variant of the benchmark are that the volumes of all tanks are non-negative: $\forall i \in \{1, \ldots , N\} : x_i \ge 0$. We consider variants where the aggregate out flow rate equals the in flow rate, so the sum of the flow rates out of all tanks equals the inlet flow rate: $w = \sum _{i=1}^N v_i$. Hence, the total volume is constant, so for all $t \ge 0$:

$$\begin{aligned} \sum _{i=1}^N x_i(t) = \sum _{i=1}^N x_i(0). \end{aligned}$$

In these instances, the purpose of the inlet is to effectively move net volume between tanks, and the search problem is to find an appropriate order of such moves to reach a specific volume level in all of the *N* tanks.

**Table 4:** Results for the tank benchmarks

Inst.	*N*	Top.	Uninformed DFS			Box-heuristic			PDB		
			#It	Length	Time	#It	Length	Time	#It	Length	Time (time abs.)
1	3	C	4	3	254.032	4	3	305.309	4	3	**166.516** (6.285)
2	3	C	4	3	237.33	4	3	238.311	4	3	**124.195** (4.71)
3	3	C	4	3	483.083	4	3	**479**.**847**	4	3	524.132 (41.855)
4	3	C	n/a	n/a	OOT	5	2	828.846	2	1	**190.139** (7.856)
5	3	C	6	5	508.693	8	2	76.68	3	2	**32.141** (11.351)
6	3	C	n/a	n/a	OOT	5	2	312.54	3	2	**220.108** (9.272)
7	3	L	6	5	506.076	5	2	281.351	3	2	**218.484** (7.791)
8	3	L	3	2	280.473	3	2	**276**.**498**	3	2	289.545 (6.156)
9	4	L	n/a	n/a	OOT	5	4	**6**.**171**	13	5	24.972 (8.688)
10	3	L	6	5	270.648	5	2	144.673	2	1	**41.345** (0.84)
11	4	L	18	6	30.95	11	6	23.81	4	3	**8.696** (3.199)
12	5	L	10	7	23.949	14	7	63.518	4	3	**18.138** (9.289)
13	6	L	39	27	64.091	28	21	51.595	4	2	**21.208** (6.283)
14	7	L	53	34	130.636	44	22	**117**.**732**	9	5	117.763 (9.536)
15	8	L	37	29	**108.7**	46	21	164.349	33	29	140.968 (30.271)

Bold values indicate the best analysis method for every benchmark

*N*: number of tanks (numbers of locations #loc and continuous variables), *Top* topology (*C* complete, *L* linear), *OOT* out of time (max 30 min), *Uninformed DFS* uninformed depth-first search, *Box-heuristic* box-based distance heuristic, *PDB* our PDB cost function $$ cost ^{ PP }$$, *#loc* number of locations, *#it* number of iterations, *length* length of the found error trajectory, *time* total time in seconds including any preprocessing

**Fig. 17:**
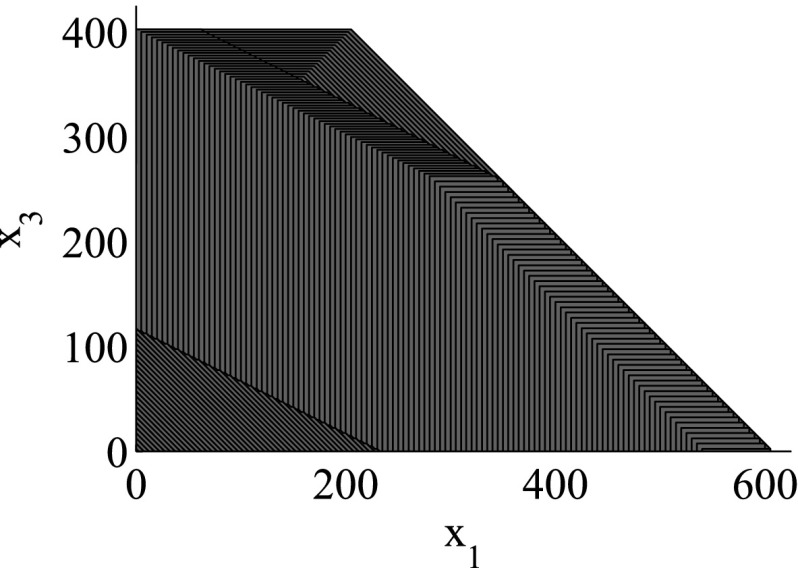
*Water tank benchmark*: Uninformed search error trajectory for instance 10

**Fig. 18:**
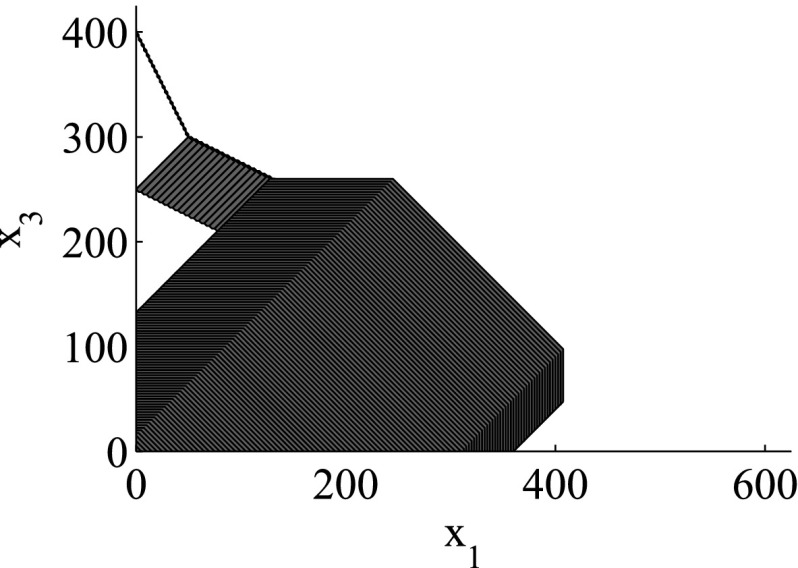
*Water tank benchmark* box-based heuristic search error trajectory instance 10

**Fig. 19:**
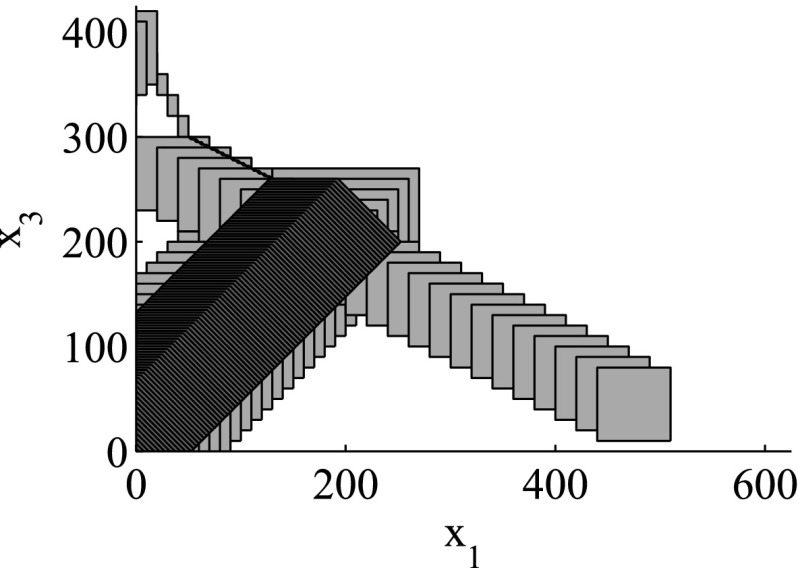
*Water tank benchmark* PDB search error trajectories instance 10 (abstract: *light gray*, concrete: *dark gray*)

The results for the water tank problem class are provided in Table 4. Again, the results are similar to the results in the navigation and the satellite benchmark classes: We observe that PDBs can help significantly in guiding the search towards error states. For example, comparing Figs. 17, 18, and 19, which each, respectively, show an execution of uninformed search, the box-based heuristic, and PDBs, we observe that our PDB-based approach is able to exploit the abstract run to more quickly find the correct sequence of tanks to fill to reach a certain region of the state-space (again, the light gray regions are covered in the abstract run only and can be computed efficiently). Generally, PDBs can particularly help for the water tank problems because of the non-determinism that occurs in this problem class (which is important to be resolved accurately, corresponding to the choice of which tanks to fill in which order). However, we also observe that in 4 cases, the overall runtime is higher than the runtime with the box-based heuristic. In these cases, the precomputation of the PDB does not pay off—we will discuss such cases in more detail below.

**Table 5:** Results for the heater benchmarks

Inst.	#loc	Uninformed DFS			Box-heuristic			PDB		
		#It	Length	Time	#It	Length	Time	#It	Length	Time (time abs.)
1	4	7	6	**148.4**	12	6	212.651	7	6	149.117 (0.625)
2	4	9	8	305.21	5	0	146.311	1	0	**10.506** (7.982)
3	4	4	$\infty$	27.476	4	$\infty$	27.579	4	$\infty$	**27.467** (0.042)
4	4	7	$\infty$	178.781	7	$\infty$	177.748	7	$\infty$	**176.566** (1.1)
5	4	21	20	**84.779**	n/a	n/a	OOT	21	20	98.173 (12.303)
6	4	4	1	**1.284**	n/a	n/a	OOT	4	1	1.705 (0.4)
7	4	4	2	34.493	4	2	34.525	3	1	**32.07** (3.148)
8	4	7	6	**89.724**	49	48	907.52	7	6	90.778 (0.475)
9	4	4	2	8.772	3	2	**8.183**	3	1	8.28 (4.687)
10	4	5	4	**27.164**	15	8	65.249	5	4	27.851 (0.635)
11	4	13	8	25.771	25	14	48.844	12	8	**23.708** (0.435)
12	4	3	0	10.603	3	0	10.601	2	0	**8.212** (0.544)
13	4	n/a	n/a	OOT	n/a	n/a	OOT	10	6	**640.441** (240.583)
14	4	7	6	**58.533**	36	22	284.592	7	6	59.157 (0.55)
15	4	9	8	**38.06**	42	24	150.263	9	8	41.948 (3.752)

Bold values indicate the best analysis method for every benchmark

*OOT* out of time (max 30 min), *Uninformed DFS* uninformed depth-first search, *Box-heuristic* box-based distance heuristic, *PDB* our PDB cost function $$ cost ^{ PP }$$, *#loc* number of locations, *#it* number of iterations, *length* length of the found error trajectory, *time* total time in seconds including any preprocessing

### Results for heater benchmarks

This benchmark consists of variants of the heater benchmark [21]. In our variation, we consider three rooms with one heater. The automaton is modeled with four locations, consisting of no heaters on in any room, or the heater is on in one of the three rooms. The size of the problem instances have 4 locations, and all instances feature 3 temperature variables, 1 time variable, and 16 real constants. The temperature dynamics are linear and there is coupling between temperatures in different rooms. If the heater is on in a room, its temperature rate of change has a positive additive term $c_i$, but otherwise does not, so the temperature may decrease (subject to the temperatures in different rooms). Specifically, for room 1 (and symmetrically rooms 2 and 3), if the heater is on, the dynamics are: $\dot{x}_1 = b_1(u-x_1) + a_{1,2}(x_2-x_1) + a_{1,3}(x_3-x_1) + c_1$, but if the heater is off, the dynamics are the same except without the $c_1$ term. In our variant, the invariants specify only that the temperatures are all non-negative and bounded. The heater may be turned on in room $i \in \{1,2,3\}$ if $x_i \le T_{on}$ for some real threshold $T_{on}$, and turned off if $x_i \ge T_{ off }$ for some real threshold $T_{ off }$. There is non-determinism in choosing to turn off or on the heater once the threshold condition is met, and there is a potential delay in changing the state of the heater from off to on and vice-versa.

The results for the heater benchmark are provided in Table 5. We observe that, unlike the results for the other benchmarks, the results for the heater are more diverse. While the PDB approach overall performs best in 7 out of 15 problem instances, it is somewhat slower than uninformed DFS in other 7 instances. Having a closer look, we observe that the error trajectories with DFS are found with equally many iterations by SpaceEx, and additionally, their length is the same compared to those found with the PDB approach. In such cases where the PDB cannot improve over the search behavior of DFS, DFS is naturally more efficient because of the PDB’s computational overhead (in fact, the difference in search time is almost exactly due to this overhead). However, obtaining such an informed search behavior with DFS is rather based on having good luck, whereas PDBs provide a more principled approach to achieve this. Furthermore, despite the sometimes higher runtimes in this benchmark class, we observe that our PDB approach is able to solve one more problem than DFS, and three more problems than the box-based heuristic within our time limit of 30 min. In addition, similar to the satellite benchmarks, we have been able to effectively prove the absence of errors in two cases (heater instance 3 and instance 4).

**Fig. 20:**
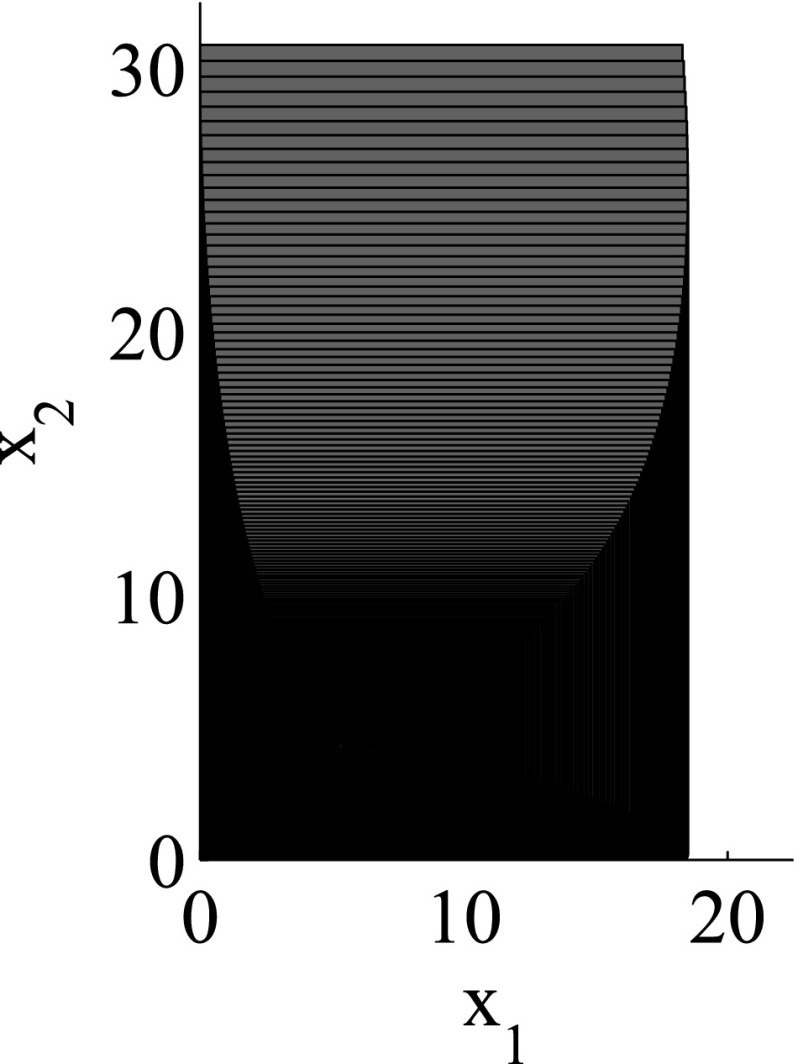
*Heater benchmark* Uninformed search error trajectory for for instance 2

**Fig. 21:**
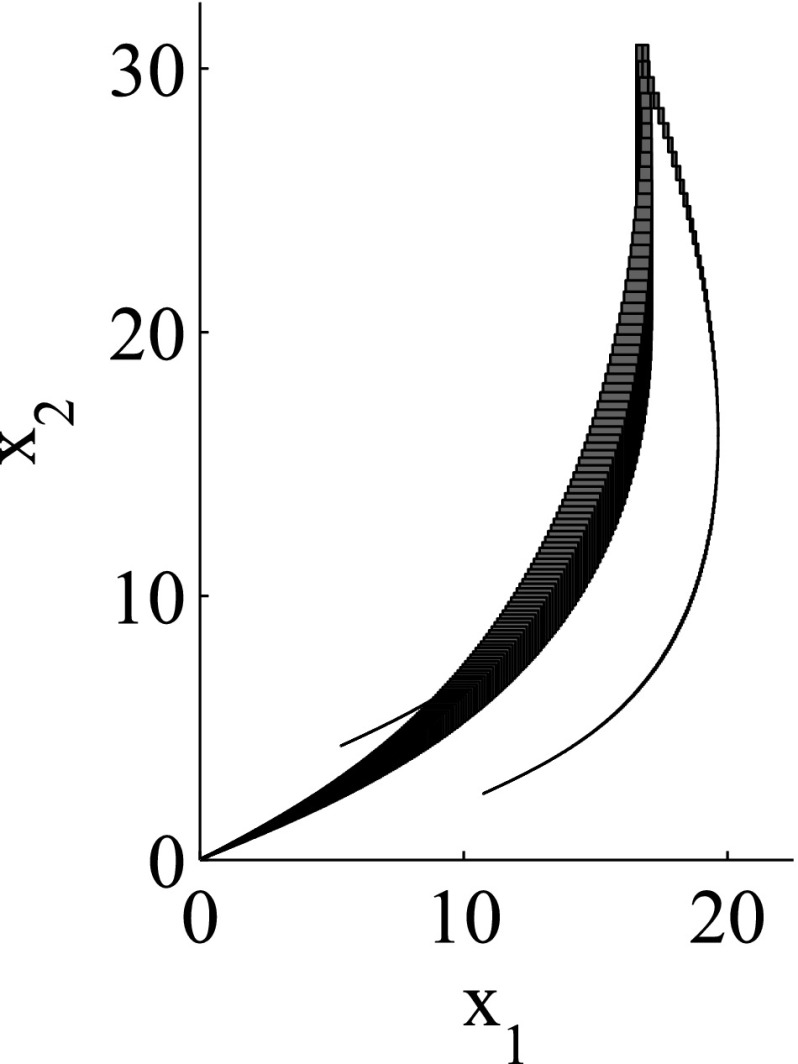
*Heater benchmark* box-based heuristic search error trajectory for instance 2

**Fig. 22:**
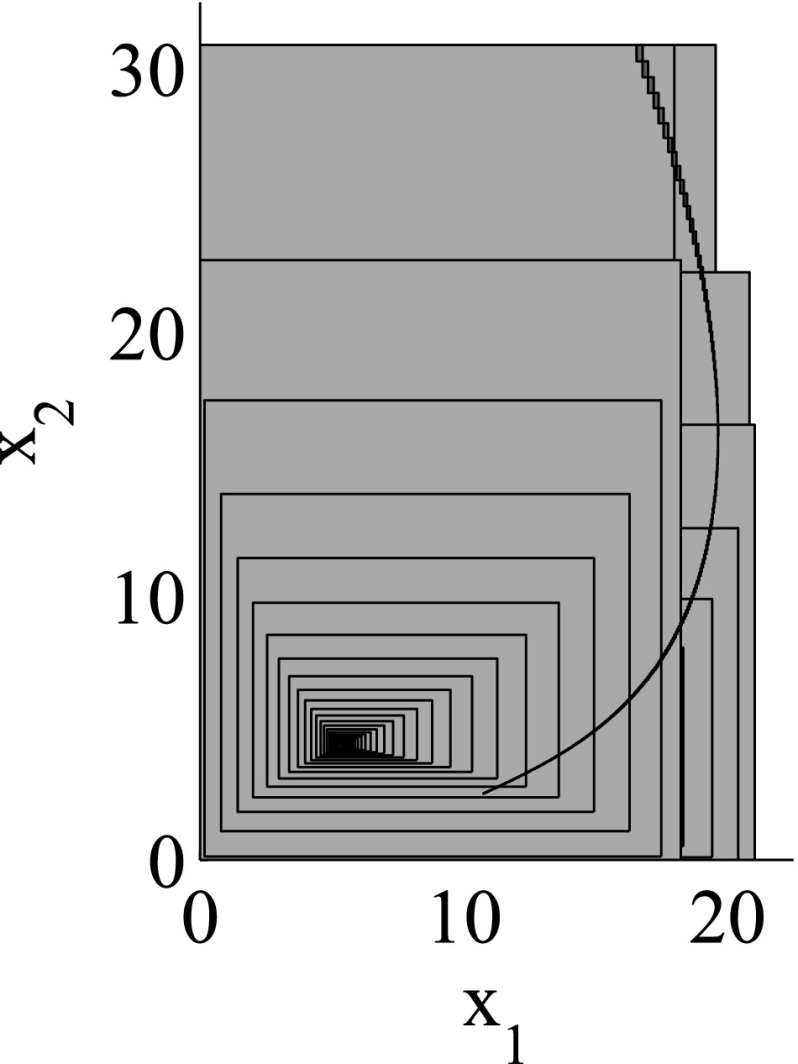
*Heater benchmark* PDB search error trajectories for instance 2 (abstract: *light gray*, concrete: *dark gray*)

Finally, for the last time, let us have a look at the covered region space by DFS, by the box-based heuristic and by PDBs in Figs. 20, 21, and 22, respectively. We observe that the concrete run with PDBs (indicated in dark gray) boils down to a small curve in this instance, whereas the other approaches cover a (much) larger fraction.

### Runtimes of partial PDBs versus full PDBs

Considering the runtime to build the partial PDBs compared to computing full PDBs, we observed that strong reductions of several orders of magnitude can indeed be obtained. In particular, the computation of the full abstract state space sometimes exceeds our time bound of 30 min, whereas the partial PDBs can still be computed efficiently. This happens in the water tank problem class, where full PDBs could not be computed within 30 min in any instance, whereas partial PDBs could be computed within less than a minute in all of the 15 instances (less than 10 s in 9 of these instances). In this respect, we conclude that the notion of partial PDBs particularly makes the overall approach tractable on a larger class of problems. In cases where full PDBs can be computed within 30 min, the runtime can be significantly higher than with partial PDBs: For example, in the satellite domain instance 10, computing the full PDB needs around 175 s, compared to roughly 3 s for computing the partial PDB.

### Discussion

We have observed that PDBs can provide more informed search behavior than uninformed search or than the box-based heuristic. A potential problem is the computational overhead due to its precomputation time. We will discuss advantages and drawbacks of our PDB approach in this section.

As a general picture, we first observe that the number of iterations of SpaceEx and also the length of the found error trajectories are mostly at most as high with PDBs as with uninformed search and the box-based heuristic. In particular, our PDB approach could solve several problem instances where uninformed search and the box-based heuristic ran out of time. In some cases, the precomputation of the PDB does not pay off compared to DFS and the box-based heuristic – however, in such cases, the pure concrete search time with PDBs is still mostly similar to the pure search time of DFS and the box-based approach.

We further observe that the length of the trajectories found by the box-based heuristic and the PDB heuristic is often similar or equal, while the number of iterations is mostly decreased. This again shows that the search with the PDB approach is more focused than with the box-based heuristic in such cases, and less backtracking is needed. In particular, the box-based heuristic always tries to find a “direct” trajectory to an error state, while ignoring possible obstacles. Therefore, the search can get stuck in a dead-end state if there is an obstacle, and as a consequence, backtracking becomes necessary. Furthermore, the box-based heuristic can perform worse than the PDB if several bad states are present. In such cases, the box-based heuristic might “switch” between several bad states, whereas the better accuracy of the PDB heuristic better focuses the search towards one particular bad state. In problems that are structured more easily (e. g., where no “obstacles” exist and error states are reachable “straight ahead”), the box-based heuristic might yield better performance because the precomputation of the PDB does not pay off.

Finally, a general advantage of PDBs compared to the box-based heuristic which we did not discuss in detail so far is the broader applicability of PDBs. By definition, the box-based heuristic estimates distances by computing Euclidean distances between the region of the current and the error state. However, in problems where error states are defined solely by (discrete) locations, there is no such error region, and the box-based distance heuristic is not effectively applicable. In contrast, PDBs are more general, and applicable for all kinds of error states.

## Conclusion

We have explored the application of coarse-grained space abstractions to compute PDBs for hybrid systems. For a given safety property and hybrid system with linear dynamics in each location, we compute an abstraction by coarsening the over-approximation SpaceEx computes in its reachability analysis. The abstraction is used to construct a PDB, which contains abstract symbolic states together with their abstract error distances. These distances are used in guiding SpaceEx in the concrete search. Given a concrete symbolic state, the guiding heuristics returns the smallest distance to the error state of an enclosing abstract symbolic state. This distance is used to choose the most promising concrete symbolic successor. In our implementation, we have taken advantage of the SpaceEx parametrization support, and were able to report a significant speedup in counterexample detection and even for verification. Our new PDB support for SpaceEx can be seen as a non-trivial extension of our previous work on guided reachability analysis for hybrid systems where the discrete system structure was ignored completely [14]. For the future, it will be interesting to further refine and extend our approach by, e. g., considering even more fine grained abstraction techniques, or by combinations of *several* abstraction techniques and therefore, by combining several PDBs. We expect that this will lead to even more accurate cost functions and better model checking performance.
